# Statistically derived asymmetric membrane potentials from α-helical and β-barrel membrane proteins

**DOI:** 10.1038/s41598-018-22476-6

**Published:** 2018-03-13

**Authors:** Julia Koehler Leman, Richard Bonneau, Martin B. Ulmschneider

**Affiliations:** 1 Center for Computational Biology, Flatiron Institute, Simons Foundation, New York, 10010 NY USA; 2 Department of Biology, Center for Genomics and Systems Biology, New York University, New York, 10003 NY USA; 3 Department of Computer Science, New York University, New York, 10012 NY USA; 4 Medical School, University of Exeter, Exeter, EX1 2 LU UK

## Abstract

Modeling membrane protein (MP) folding, insertion, association and their interactions with other proteins, lipids, and drugs requires accurate transfer free energies (TFEs). Various TFE scales have been derived to quantify the energy required or released to insert an amino acid or protein into the membrane. Experimental measurement of TFEs is challenging, and only few scales were extended to depth-dependent energetic profiles. Statistical approaches can be used to derive such potentials; however, this requires a sufficient number of MP structures. Furthermore, MPs are tightly coupled to bilayers that are heterogeneous in terms of lipid composition, asymmetry, and protein content between organisms and organelles. Here we derived asymmetric implicit membrane potentials from β-barrel and α-helical MPs and use them to predict topology, depth and orientation of proteins in the membrane. Our data confirm the ‘charge-outside’ and ‘positive-inside’ rules for β-barrels and α-helical proteins, respectively. We find that the β-barrel profiles have greater asymmetry than the ones from α-helical proteins, as a result of the different membrane architecture of gram-negative bacterial outer membranes and the existence of lipopolysaccharide in the outer leaflet. Our data further suggest that pore-facing residues in β-barrels have a larger contribution to membrane insertion and stability than previously suggested.

## Introduction

Transfer free energy or hydrophobicity scales are important to understand protein folding, stability, membrane insertion, and assembly in the bilayer. For many years, researchers have derived hydrophobicity scales with different methods^1,2^, typically aiming for a universal scale that allows for identification of MPs from sequences^1,3^ as well as estimating MP topology^4^, stability of the native state, and provide an energy function for protein folding studies, optimization of protein embedding in the membrane^5^, structure prediction^6–8^, modeling^9,10^, or protein design^7,11,12^. While these studies were broadly successful at identifying hydrophobic segments, they are not accurate enough for universal prediction of MP assembly and native state structure. This is chiefly due to the enormous complexity, heterogeneity, and diversity of biological bilayers. Cellular membranes differ vastly in type, composition, and asymmetry of their lipids, as well as amount and architectures of their embedded proteins^13^. These differences are not restricted to the cell membranes between the different branches of life or organisms (eukaryotes, bacteria, archaea), but even occur within the same cell (e.g. inner and outer membranes of gram-negative bacteria, and cell organelles in eukaryotes). Many of these membranes are directly related to specific protein architectures (e.g. α-helical bundles vs. β-barrels). Thus, the goal of a universal hydrophobicity scale may be unachievable as each membrane type has its own characteristics. Hydrophobicity scales further depend on the method used for derivation (experimental or statistical), the types of proteins studied (soluble proteins, α-helical vs. β-barrel MPs) and the assumptions made about the membrane (symmetric vs. asymmetric)^1^.

Experimental derivation of hydrophobicity scales through MP folding and insertion studies is challenging. First, almost all α-helical MPs require the membrane-embedded translocon for insertion^14^. While some outer membrane β-barrels are able to insert spontaneously into lipid vesicles^15^, the majority of them require the β-barrel assembly machinery (BAM) for insertion *in vivo*^16,17^. The exact workings of these molecular machineries are currently unknown. Second, extraction of thermodynamic parameters requires an equilibrium experiment that drives the system between folded and unfolded states and for which the system moves cyclically along the same pathway in opposite directions, i.e. their folding/unfolding curves must overlay without hysteresis^18^. Third, de- and renaturation of different proteins typically happen under distinct experimental conditions that have to be tested individually: while acid- and thermal denaturation seem to incompletely denature secondary structure in most MPs^19^, chemical denaturation by guanidine hydrochloride, urea, or SDS was used successfully for a number of α-helical and β-barrel MPs^19^. However, for these systems the unfolded reference state is unnatural, as these approaches only yield relative free energy differences for mutated residues.

Since reversible (un)folding and insertion of MPs remains difficult, only few hydrophobicity scales were derived by experimental means. For the β-barrel outer membrane phospholipase A (OmpLA), a sidechain hydrophobicity scale was derived from reversible (un)folding into lipid vesicles, using GdnHCl as a chemical denaturant^20^, and carefully adjusting experimental parameters to circumvent folding hystereses. This work was recently expanded to measure depth-dependent transfer free energies for the aromatic amino acids^21^ - we review these and other depth-dependent profiles in Table 1. For α-helical proteins, an apparent (or ‘biological’) hydrophobicity profile was derived experimentally by inserting a transmembrane segment into the leader peptidase protein, into which all amino acids were introduced at different depths^22^. Recently, an asymmetric potential was derived for insertion of a single TM helix using a mutational scanning approach^12^. A large number of mutations were tested in a high-throughput manner via protein sequence libraries, for which levels of TM span expression, insertion, and association were measured using orthogonal antibiotic resistance markers. With this clever approach, high-resolution structural models of TM helix dimers could be computed from homology models and the obtained TM association data^12^.

**Table 1:** Symmetric and asymmetric depth-dependent potentials derived from experimental data or statistics.

**author**	**year**	**α/β** ^**a**^	**E/S** ^**b**^	**S/A** ^**c**^	**ref**	**comments**	**#prot** ^**d**^	**embedding** ^**e**^	**SASA** ^**f**^	**bin size**	**functional form**
Hessa & von Heijne, biological scale	2007	α	exp	sym	^22^	Inserted a TM segment into the leader peptidase protein, into which all amino acids were introduced at different depths. Translocation into microsomes was quantified by the number of glycosylation sites on either terminus of the TM segment. 324 19-residue TM segments were measured to compute ΔΔG’s	1	center of TM segment is at membrane center	protein is 3-helix bundle, so most are lipid-exposed	residue	double Gaussian for WY, single Gaussian for others
Elazar & Fleishman, dsTbL	2016	α	exp	asym	^12^	Combined sequence libraries with TOXCAT assay in whole cells. Measured TM span expression, insertion and association depending on residue depth with orthogonal antibiotic resistance markers. 472 mutants were tested 100 times each in a high-throughput manner to compute ΔΔG’s	1	membrane center was estimated by aligning ILMF profiles’ troughs	single helix, so all lipid-exposed	residue	4 D polynomials
Senes & DeGrado, Ez potential	2007	α	stat	sym	^23^	statistical potential derived from 24 MPs in a symmetric manner; insufficient counts for C	24	protein COM at membrane center	no distinction	2 Å	double Gaussian for WY, sym sigmoidal for others
Ulmschneider, implicit membrane potential	2005	α	stat	asym	^33^	statistical potential derived from 46 MPs in an asymmetric manner; resolution ≤4 Å; insufficient counts for CST;	46	centered DSSP TM spans at membrane center	SASA probe radius 1.4 Å	2 Å	double Gaussian for RKDEHWY, single Gaussian else
Schramm & DeGrado, Ez potential	2012	α	stat	asym	^25^	statistical potential derived from 76 MPs in an asymmetric manner; sequence similarity ≤30%, resolution ≤ 3.5 Å	76	OPM embedding	SASA probe radius 1.9 Å	2 Å	sigmoid, Gaussian or combination of the two
this work	2018	α	stat	asym	this	statistical potential derived from 239 MPs in an asymmetric manner; sequence similarity ≤30%, resolution ≤3 Å	239	topology from OPM but embedding from PDBTM	lipid-exposed vs buried^35^	3 Å	double Gaussian
Moon & Fleming, sidechain hydrophobicity scale	2011	β	exp	sym	^20^	reversible GnHCl (un)folding of OmpLA into DLPC vesicles to derive symmetric profile; an A residue at the membrane center was mutated into all 19 other amino acids; 3 titrations for WT and 2 titrations for mutants to compute ΔΔG’s; potential derived for LR	1	membrane center set halfway between aromatic girdles	only lipid-exposed residues	residue	single Gaussian for LR, no fit parameters given
MacDonald & Fleming, sidechain hydrophobicity scale	2016	β	exp	sym	^21^	reversible GnHCl (un)folding of OmpLA into DLPC vesicles; residues at different depths were mutated into WYF; 3 titrations for WT and 2 titrations for mutants to compute ΔΔG’s;	1	center from MD simulations: COM of the protein and phosphate atoms	only lipid-exposed residues	residue	linear for WYF
Hsieh & Nanda, Ez potential	2012	β	stat	sym	^26^	statistical potential derived from 35 MPs in a symmetric manner; sequence similarity ≤26%; insufficient counts for CM	35	embedding from OPM TM spans	SASA > 0.2	3 Å	double Gaussian for WYFG, sym sigmoid for others
Wimley	2002	β	stat	asym	^54^	statistical 3-state hydrophobicity scale from 15 non-redundant β-barrels;	15	aromatic girdles were used	lipid vs water exposed	regions	no fitting done
Jackups & Liang, positive outside rule	2006	β	stat	asym	^27^	sequence similarity ≤ 26%; resolution ≤2.6 Å; derived statistics for regions, depending on z and burial, but no depth-dependent potential like the others, they use it to derive a basic energy function for barrel prediction based on H-bonds	19	embedding from OPM TM spans	regions	regions	no fitting done
Slusky & Dunbrack, charge outside rule	2013	β	stat	asym	^15^	statistical potential derived from 55 MPs in an asymmetric manner; sequence similarity ≤50%; resolution ≤3.5 Å; only averages of AA groups were fit, but not individual AA types; insufficient counts for PCMT	55	N/C termini are inside, membrane center defined where phospholipid meets LPS and aromatic girdle set to −12Å	lipid vs water exposed	3 Å	RKDE to P2, NQHS to P2, AGILV to P2, FWY to P4, no fitting parameters given
Lin & Liang, TMSIP	2017	β	stat	asym	^28^	19 MPs were used to derive an energy function that includes a membrane burial term and inter- and intra-strand H-bond interaction energies^27^, the energy function was used to derive a statistical potential for ΔΔG’s tested on 24 MPs; sequence similarity ≤26%; resolution ≤2.6 Å	19	embedding from OPM TM spans	lipid vs water exposed	residue (regions for deri- vation)	double Gaussian for WY, single Gaussian for others
this work	2018	β	stat	asym	this	statistical potential derived from 96 MPs in an asymmetric manner; sequence similarity ≤50%, resolution ≤3 Å; insufficient counts for C	96	topology from OPM but embedding from PDBTM	lipid-exposed vs buried^35^	3 Å	4D polynomial for IM, double Gaussian for others

^a^α-helical or β-barrel.

^b^Experimental or statistical.

^c^Symmetric or asymmetric.

^d^number of proteins used for derivation.

^e^How proteins were embedded/centered in the membrane.

^f^was distinction made between lipid-exposed and lipid-buried residues or how was SASA calculated.

Since experimental measurement of MP folding, insertion, and association remains challenging, known protein structures were used to derive statistical hydrophobicity scales, which were later expanded to depth-dependent potentials (Table 1). For α-helical proteins, Senes, DeGrado and co-workers derived a symmetric knowledge-based potential^23^, which the authors termed the *Ez* potential. In accordance with the positive-inside rule for helical proteins in the inner membrane^24^, an asymmetric potential was derived by Ulmschneider *et al*. In 2012, the *Ez* potential was adjusted for asymmetry from a larger database than previous^25^. Symmetric and asymmetric hydrophobicity profiles were also derived from outer membrane β-barrels. In 2012, a symmetric *Ez* potential was derived^26^, even though a decade earlier, Wimley extracted asymmetric amino acid distributions from 15 β-barrels. Jackups and Liang confirmed the compositional bias of positive charges on the outside of the outer membrane, and termed this the ‘positive-outside rule’^27^. In 2013, Slusky and Dunbrack broadened this observation to the ‘charge-outside rule’^15^. This finding was supported recently by another asymmetric potential from Liang and co-authors^28^, which was used to orient β-barrels in asymmetric bilayers.

One of the largest unsolved problems is the lack of experimental data identifying exactly how specific proteins are embedded in a lipid bilayer. The membrane mimetics used for structural studies (micelles, bicelles, lipidic cubic phases, nanodiscs) are typically far from planar bilayers, complicating the observation of proper membrane embedding. Further, only few structures contain individual lipids and protein embedding likely depends on acyl chain length, lipid composition or even specifically bound lipids. Scientists have thus derived scoring functions to predict protein embedding in the membrane; however, lack of benchmarking data precludes extensive testing of these methods. An earlier method used by the PDBTM database is TMDET^29^, which cuts the protein into slices and uses hydrophobicity and structural information of the Cα-trace (such as straightness, turns, and termini) to derive an objective function, which is used to optimize protein embedding. While inspection of protein embedding over a database confirms good results, protein in/out topologies in PDBTM are inconsistently handled. A more recent method that coherently accounts for topology is the PPM server used by the OPM database^30^. While OPM is widely used also for derivation of other methods, comparison of protein embedding for larger databases between PDBTM and OPM indicates inferior embedding for the latter.

Here, we have derived implicit membrane potentials from amino acid distributions in both β-barrel and α-helical MPs. The potentials are derived in an asymmetric manner to distinguish inner and outer leaflets of two distinct membrane types: the β-barrel potential was derived from proteins embedded primarily in bacterial outer membranes that have an LPS layer, and the α-helical potential was derived from proteins mainly embedded in membranes lacking the LPS layer. Identical approaches for the derivation allows for direct comparison between these different membrane types. We distinguish between lipid-accessible and lipid-inaccessible residues for derivation. These profiles capture known effects such as the *positive-inside rule* for α-helical MPs and the *charge-outside rule* for β-barrels and indicate that pore-facing residues in β-barrel MPs play a non-negligible role in MP insertion and stability. Our potentials can recapitulate MP topologies and energy profiles for various insertion depths and tilt angles on a few examples. They can further be used as scorefunctions to model MP structures^31^ and their interactions with each other, lipids, and small molecule drugs.

## Results and Discussion

We have derived individual implicit membrane potentials from databases of β-barrel and α-helical MPs in an asymmetric fashion, i.e. distinguishing the inner leaflet (z < 0 in our Figures) from the outer leaflet (z > 0 in our Figures). We further distinguished lipid-accessible and lipid-inaccessible residues. The database of β-barrel MPs contained 96 proteins with sizes from 135 to 4067 residues and a sequence similarity cutoff of 50% (see Supplementary Methods). The database of α-helical MPs contained 239 proteins with sizes from 40 to 5319 residues and a sequence similarity cutoff of 30% (see Supplementary Methods). While it would have been advantageous to derive membrane potentials for a variety of different membrane types, the number of proteins in each bilayer type is too small to derive reliable potentials; Supplementary Table 1 shows the number of known protein structures in each membrane type according to the OPM database. These numbers are for all MP structures, i.e. they are redundant in the types of proteins determined, while the potentials were derived from non-redundant databases.

### Residue statistics exhibit higher asymmetry in β-barrel database

**Figure 1:**
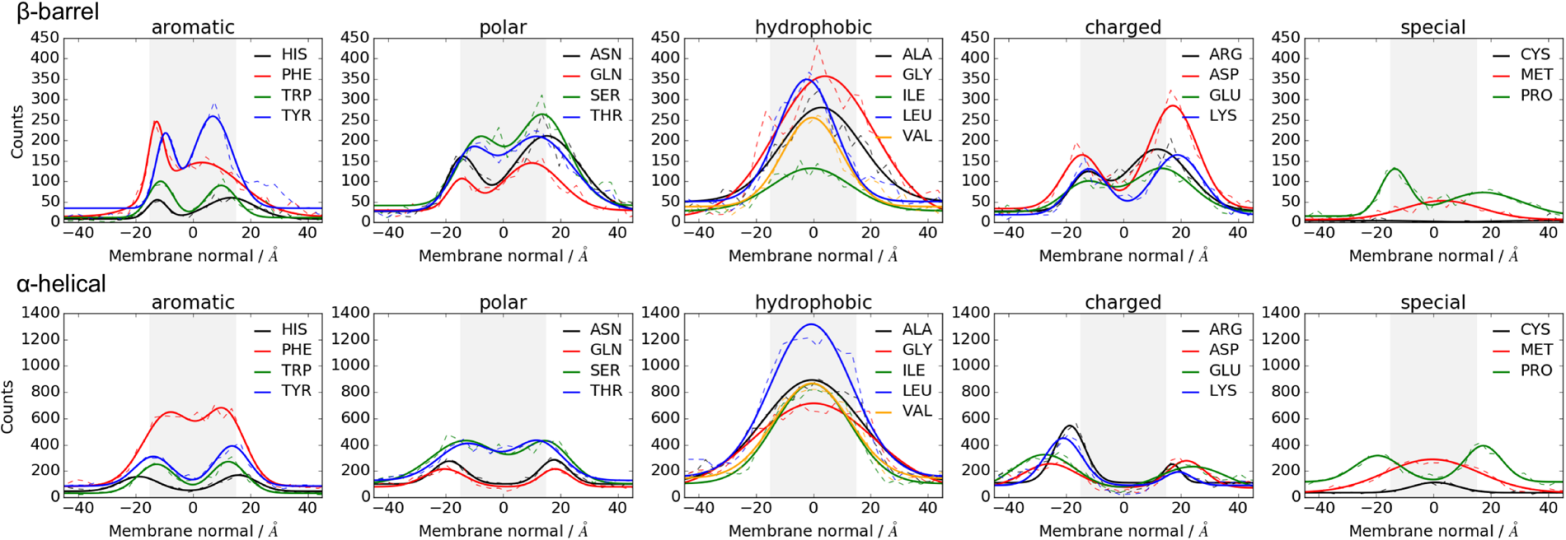
Raw counts (dashed lines) and fits (solid lines) for amino acid occurrences in β-barrel and α-helical membrane proteins. The cytoplasmic/periplasmic side is at negative numbers along the membrane normal. Each fit includes lipid-accessible and lipid-inaccessible residues (see Supplementary Figs. S1 and S2). Fitting parameters are given in Supplementary Tables 2 and 3.

As seen in Fig. 1, overall residue statistics for β-barrel MPs exhibit considerable asymmetry. Phe has higher counts at the inner leaflet and Tyr has a broader peak at the outer leaflet of the membrane interface. Thr has higher occurrences in the lipid-accessible domain than any other polar amino acid (its methyl group is more hydrophobic than for instance the hydroxyl group of Ser), while Ser is more prominent in the lipid-inaccessible (and mostly water exposed) domain, likely because the hydroxyl group can interact with water molecules in the barrel pores. For hydrophobic amino acids, Leu is more prominent in the lipid-accessible region (very hydrophobic) while Gly has higher counts for lipid-inaccessible regions, which are mostly water exposed. Gly is also the most common amino acid in the TM domain. All charged amino acids are more prominent on the outer leaflet of the membrane in agreement with the *charge outside rule*^15^. Similar as for α-helical proteins, Met is more prominent at the membrane center, while Pro occurs more often at interfacial positions, especially on the inner leaflet.

Amino acid distributions of α-helical proteins exhibit more or less symmetric distributions. From the raw counts, we can see that for the aromatics, Phe has the highest occurrence across the membrane, most of these residues are lipid-accessible. For polar amino acids, Ser and Thr have similar counts (maximum around 400 counts), as have Asn and Gln (maximum around 200 counts). From the hydrophobic amino acids, Leu occurs much more often at lipid-accessible positions than other amino acids. The distribution of the charged amino acids follows the *positive inside rule*, as Arg/Lys occur more often at the inner leaflet. Further, Arg and Lys are more prominent in the membrane than Glu or Asp and all charged amino acids avoid the center of the bilayer but are prominent at the interface. Met and Cys occur more often in the membrane center, while Pro prefers the interface.

### Implicit membrane potential for β-barrels captures the charge outside rule for outer membranes

**Figure 2:**
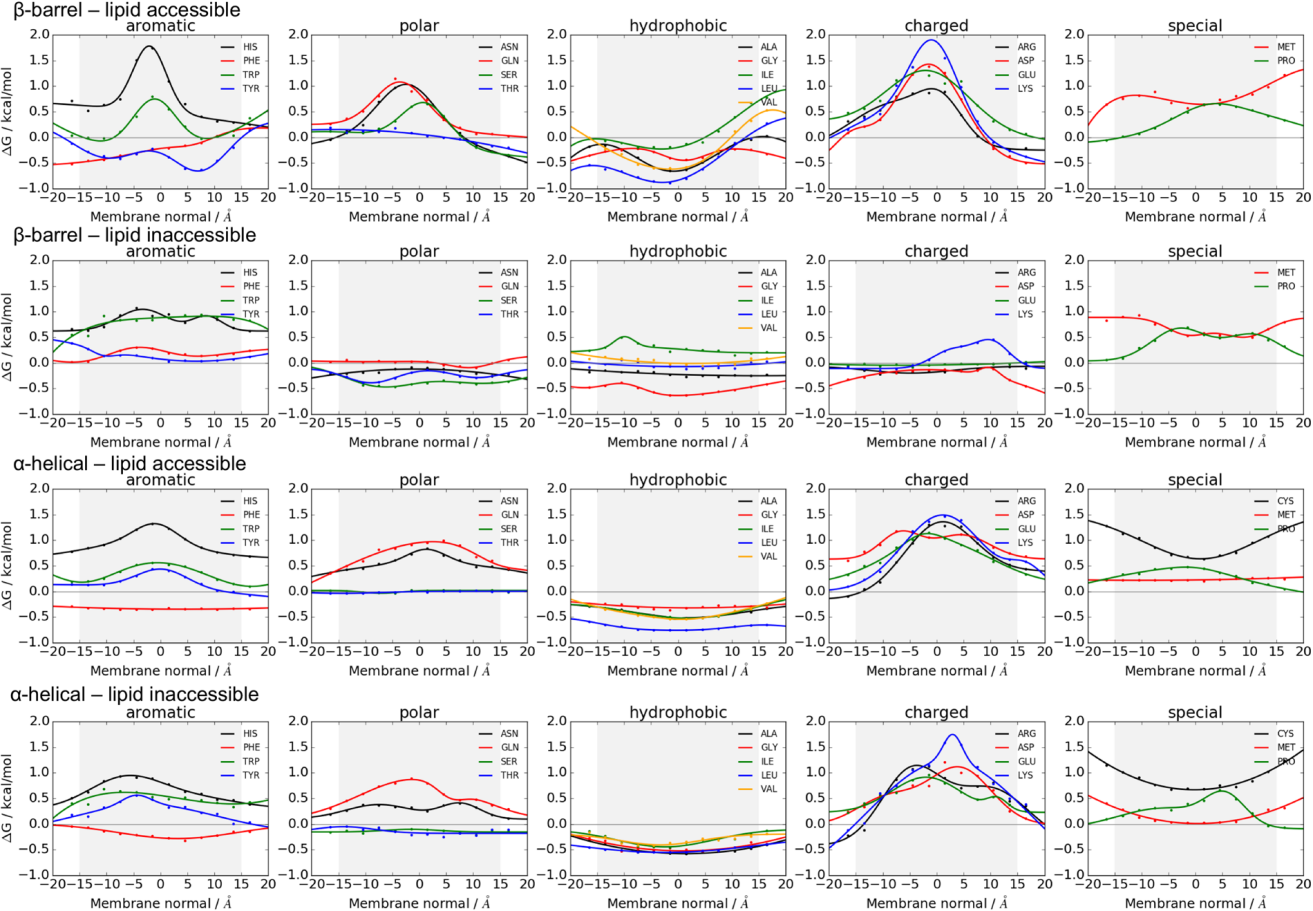
Implicit free energy profiles for lipid-accessible and lipid-inaccessible residues for β-barrel and α-helical membrane proteins. The cytoplasmic/periplasmic side is at negative z-axis along the membrane normal. Fitting parameters are given in Supplementary Tables 4–7.

When comparing profiles for lipid-accessible and -inaccessible residues for β-barrels (Fig. 2), one should note that most lipid-inaccessible residues are facing the pore, therefore being water accessible. The potentials for aromatic residues are flatter than the lipid-accessible ones and they have positive energies throughout. Polar profiles are mostly negative (favorable) while for lipid-accessible residues, they are positive. Hydrophobic profiles for lipid-inaccessible residues are more positive except for Gly which has the most negative profile of all amino acids in the lipid-inaccessible region. The profiles of the charged amino acids have small favorable energies, except Lys in the outer leaflet. Met and Pro both are unfavorable in the lipid-inaccessible regions of the protein.

**Figure 3:**
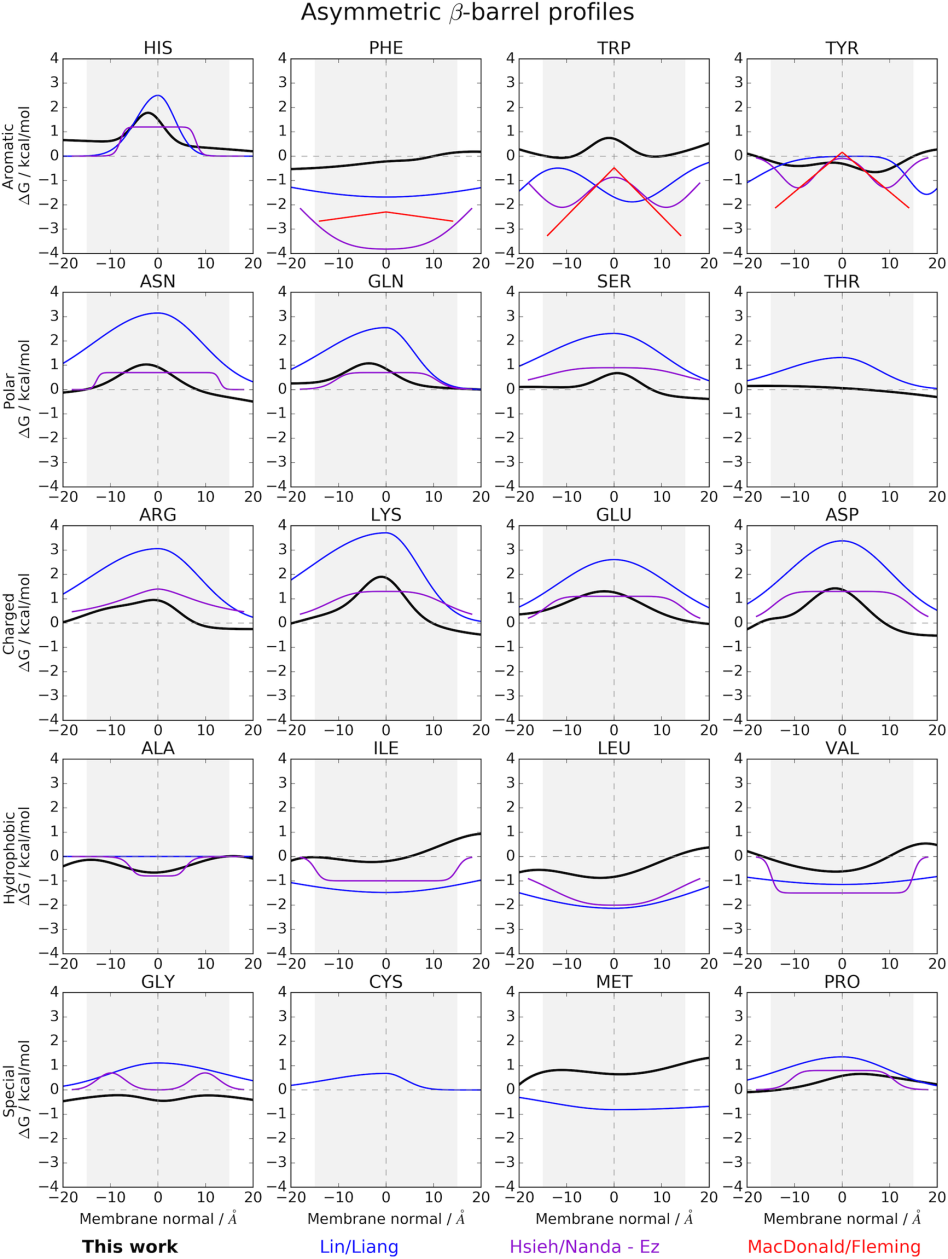
Comparison of our implicit potentials for β-barrels membrane proteins with profiles from the literature, see legend at the bottom. Details about the various profiles can be found in Table 1.

For β-barrel MPs, we can compare our implicit potential (Fig. 2) to a recent profile from Lin^28^ (Fig. 3). The main difference is that Lin and co-workers derived their potential for residue positions along β-barrels while our profile is depth-dependent in values of Å (see also Table 1). Further, Lin’s values have larger amplitudes up to 4 kcal/mol, which might stem from the fact that their potential was indirectly derived: the authors derived an energy function from 19 β-barrels^32^, which was used for mutational studies to derive the depth-dependent potential^28^. Our profiles show many similarities compared to Lin’s, yet are often shifted towards more positive or negative values, as seen for polar and charged amino acids. All charged amino acids in our potential exhibit asymmetric profiles with lower free energies at the outer leaflet of the membrane in agreement with the *charge outside rule*^15^. In our case, aromatic profiles exhibit more asymmetry for His and Phe; Phe is shifted towards more positive energies and Trp is shifted along the membrane normal. Gly and Ala are preferred at the membrane center and Ile, Leu, Val and Met are more positive in our potential. Gly and Pro are shifted towards more negative energies.

Figure 3 also shows that the *Ez* potential from Hsieh & Nanda has substantially steeper transitions between the membrane and water and is also flatter in most cases, except for Phe. Further, it is derived in a symmetric manner. These differences create a rather stark contrast between the *Ez* potential and the asymmetrically derived ones. Interestingly, there are regions in the *Ez* potential for Trp and Tyr that agree very well with the experimentally derived values from MacDonald & Fleming in the membrane region, even though the fitting functions were entirely different (double Gaussian vs. linear).

### Implicit membrane potential for α-helical bundles captures the positive inside rule

**Figure 4:**
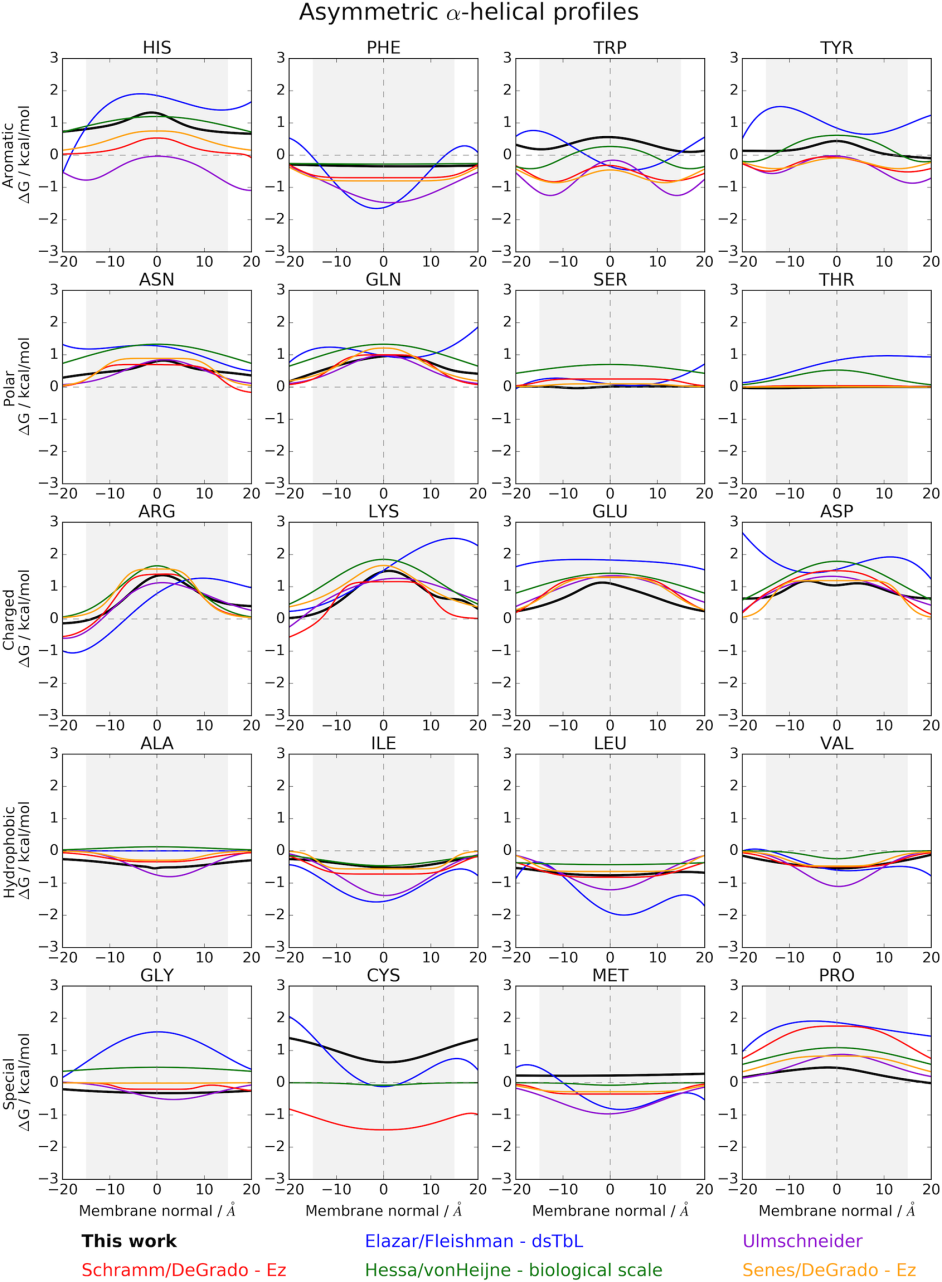
Comparison of our implicit potentials for α-helical membrane proteins with profiles from the literature, see legend at the bottom. Details about the various profiles can be found in Table 1.

As shown before, implicit potentials for α-helical proteins that were derived in an asymmetric manner still exhibit mostly symmetric distributions^25,33^ (see Fig. 4 in^12^ and Figs 2–4**)**. The exception is the dsTβL helical potential that was derived from experimental residue insertion measurements on single TM helices^12^. For helical proteins, our implicit membrane potential for lipid-accessible residues is generally in good agreement with previous studies^23,25,33^ (see Fig. 4 in^12^), even though some of the amino acids are shifted towards more positive or more negative values. Our aromatic potentials agree very well with von Heijne’s biological scale, while our polar profiles agree well with either DeGrado’s *Ez* or Ulmschneider’s potential. The largest asymmetries for α-helical proteins are seen for Arg and Lys in agreement with the positive inside rule^34^. Generally, our profiles for the charged amino acids agree well with the other potentials, even if there are slight differences in functional form or depth of potential. The profiles for the hydrophobic amino acids Ala, Ile, Leu, Val, Gly and Met are flatter than the other profiles, Met has more positive values, while Pro has more negative values. The potentials for Cys differ most due to low counts.

When comparing lipid-accessible and -inaccessible potentials for α-helical proteins (Fig. 2), we generally find good agreement. This is expected since most α-helical proteins are folded bundles without water-accessible pores. The profiles for lipid-accessible and lipid-inaccessible residues look very similar even though some of the potentials have shoulders in their distributions, for instance Tyr, Asn, Gln, Val, Arg, Glu, Asp, and Pro.

### Our implicit potentials take advantage of larger structure databases and latest methods

By examining Table 1, differences in derivation of these potentials become apparent, which likely result in different depth-dependent profiles. (1) The largest difference is between α-helical and β-barrels MPs, which reside in different types of membranes, hence different potentials were derived for them. (2) The details of the experiment (vesicles, whole cells) or statistical derivation determines whether the resulting potential is symmetric or asymmetric with respect to the membrane plane. In Figs 2–4 the inside/outside of the membrane is on the negative/positive x-axis along the membrane normal. Topology definitions for specific types of membranes can be found at http://opm.phar.umich.edu/about.php?subject = topology. (3) As mentioned above, protein embedding in the membrane is difficult to measure, yet results in shifts of the potentials along the membrane normal. As seen from Table 1, various ways to establish protein embedding have been used in derivation of these potentials. Further, databases such as PDBTM and OPM that are typically used for statistical derivation, rely on distinct algorithms with differing accuracies. In our hands, protein embedding (specified by depth in the membrane and tilt angle) from PDBTM is superior to the one from OPM, while the latter has more accessible topology information (i.e. in/out orientation). (4) Accurate potentials require distinguishing lipid-accessible vs. lipid-inaccessible residues in β-barrels and pore-forming α-helical proteins, yet only recently was an automated method developed for that purpose^35^, which we use here. Some literature potentials were derived using solvent-accessible surface area (SASA) calculators. While these distinguish solvent-exposed vs. buried residues, they don’t differentiate the type of solvent, i.e. lipid-facing vs. water-facing residues, which can distort and attenuate the resulting potentials. (5) For statistical derivation the database size can have a large effect on derived potentials, especially for small databases and rarely occurring amino acids such as Cys, Met and Pro. Our databases are 3 or 4-fold larger (for α-helical proteins and β-barrels, respectively) when compared to previous potentials. (6) Fitting functions result in variations in the potentials, particularly for sparse data. Interestingly, literature potentials were derived with a variety of fitting functions, ranging from linear, sigmoidal, single Gaussian, double Gaussian, polynomials, and asymmetric complex functions combining multiple of the aforementioned. Our potentials were fit with a functional form that led to the smallest residual, which happen to be either Gaussians or polynomial functions (see supplementary tables).

Finally, given the differences between the potentials discussed here, specific profiles might be used to best fit the problem at hand; Table 1 should help in choosing the best options. We carefully derived our potentials in a manner consistent with the latest developments in the field in terms of database size, protein embedding definitions, lipid-accessibility, and asymmetry along the membrane normal. However, we acknowledge that this might not be the best choice under certain circumstances, for instance when comparing to experimental data that were measured in a symmetric membrane environment.

### β-barrel potential properly reflects asymmetry in the bacterial outer membrane

The largest difference between the potentials for lipid-accessible residues in α-helical and β-barrel MPs is that the higher asymmetry of the β-barrel profiles. Even though the *positive inside rule* in α-helical MPs leads to an asymmetry in Arg and Lys with favorable energies at the inner leaflet, the asymmetry of all charged residues is much more pronounced in β-barrels, with all except Glu having favorable energies in the outer interface region outwards. This effect is consistent with the structure of the bacterial outer membrane, which is distinct from other membrane types. The latter are mainly composed of phospholipids and both leaflets have differing lipid compositions, leading to somewhat asymmetric biophysical properties. However, for bacterial outer membranes, the inner leaflet consists mainly of phospholipids, while the outer leaflet is composed of lipopolysaccharide (LPS) with vastly different biophysical properties. The hydrophobic core of LPS is composed of lipid A with typically six acyl chains attached to a long polysaccharide headgroup, consisting of inner core, outer core and O antigen. The number of acyl chains and structure of headgroup components vary between organisms. The inner core of the polysaccharide chain is highly negatively charged as it contains a number of phosphate groups. It also binds positively charged Ca^2+^ and Mg^2+^ ions, creating a highly charged environment. The pronounced asymmetry in the membrane potentials of charged residues with favorable energies on the outer leaflet is therefore consistent with the structure of bacterial outer membranes. [An excellent review about outer membrane structure and simulations is from Gumbart and co-workers^36^.]

### Implicit membrane potentials recapitulate topological insertion of proteins in the membrane

Our implicitly derived membrane potentials can be used to identify MP topology. We used topologies from OPM^30^ for comparison, which are obtained either from the literature or the Uniprot database^37^, which retrieves the topology from the primary literature or, if unknown, uses the sequence-based TMHMM topology predictor^38^. For scoring topologies, we only considered residues in the membrane, yet distinguished lipid-accessible and –inaccessible residues. Each residue was assigned a free energy score, depending on the amino acid type and therefore the functional form (see Supplementary Tables 4–7), its lipid accessibility, and the depth of the residue as obtained from its Cα atom. The scores over all residues were then summed to a total score; the lower the score, the more energetically favorable the inserted topology.

**Figure 5:**
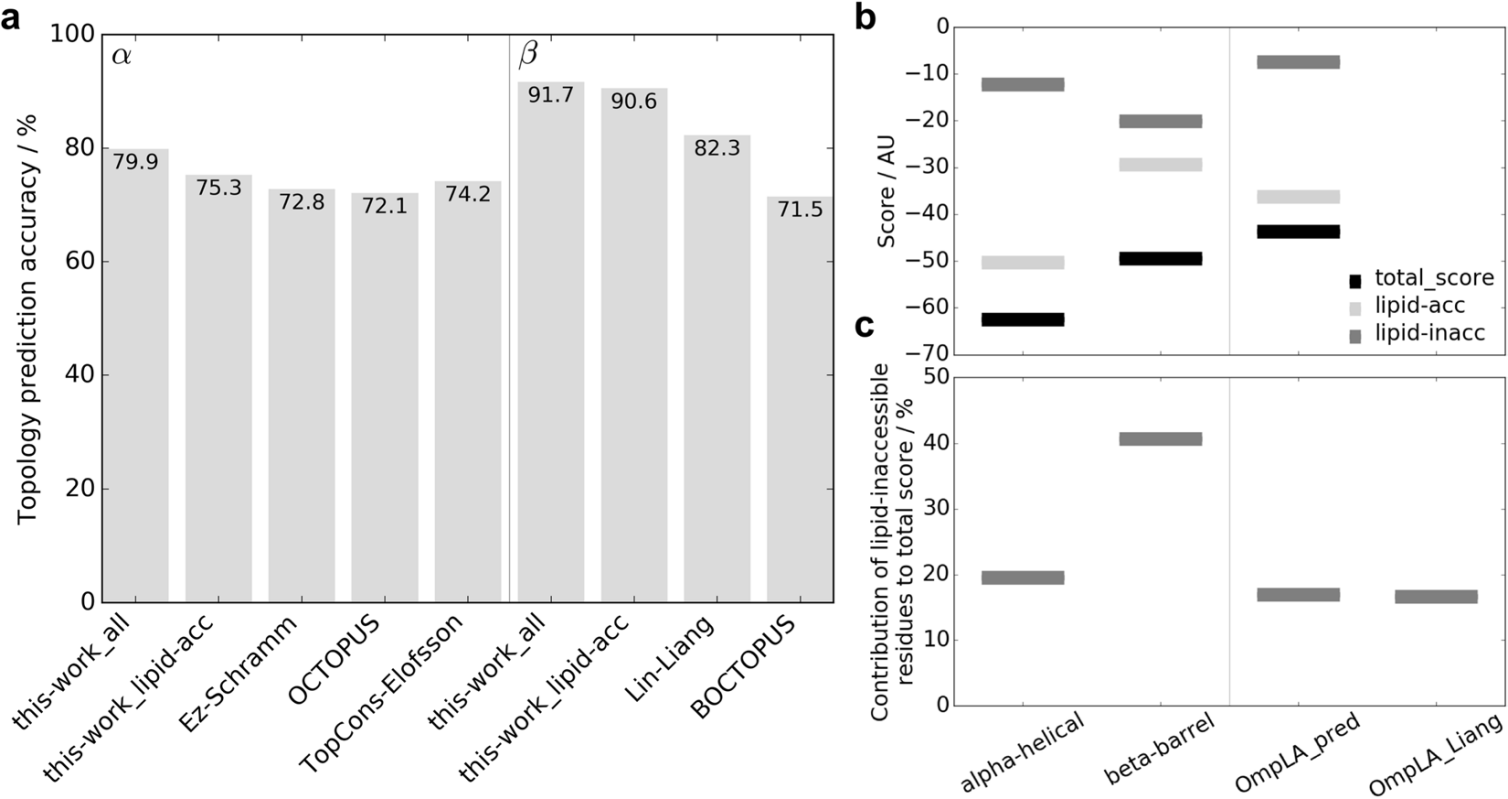
(**A**) Prediction accuracies of different topology predictors in percent for both α-helical and β-barrel proteins. We compute prediction accuracies from residues in the membrane for both lipid-accessible and lipid-inaccessible residues (denoted ‘all’) and for lipid-accessible residues only (denoted ‘lipid-acc’) for a fair comparison to other methods. Note that OCTOPUS, TopCons and BOCTOPUS are sequence-based machine learning methods. Details about the different methods and accuracies are given in the results section. (**B**) Average free energy scores for the native position (position (0, 0) in Fig. 7) over all α-helical (239 proteins) and β-barrels (96 proteins) in our databases, as well as for OmpLA based on our prediction. The scores for the lipid-accessible residues (light gray) and lipid-inaccessible residues (dark gray) give rise to the total score (black). (**C**) Contribution of lipid-inaccessible residues to the total score. For α-helical proteins, lipid-inaccessible residues contribute on average about 19.57% to the total score – these residues are most often buried in the protein interior. For β-barrels, lipid-inaccessible residues contribute on average 40.70% to the overall score. Since lipid-inaccessible residues mostly face the aqueous pore in β-barrels, the contribution of pore-facing residues to overall insertion and stability is considerably higher than was previously suggested^28^. However, there is excellent agreement between the values suggested for OmpLA (right panels): Liang et al. estimated that lipid-facing residues contribute 16.67% to overall insertion, while our predicted value for OmpLA is 16.95%.

For α-helical proteins, we predict the same topology as in OPM for 79.9% (191/239) of the proteins (Fig. 5A) when considering both lipid-accessible and lipid-inaccessible residues. We also compute prediction accuracies on lipid-accessible residues only for a fair comparison with other potentials for which only values on lipid-accessible residues are available. While these values could be higher, one should consider that (1) the implicit potentials are almost symmetric for helical proteins, except for Arg and Lys in support of the *positive inside rule*; (2) we disregard the score difference between OPM and -inverted topologies; in fact, both might have a very similar score; (3) the error rate of the topology in the OPM database is unknown. For proteins without experimental data or identified topology in the literature, TMHMM^39^ might still incorrectly predict topology, the authors state an accuracy of 77.5%.

Nevertheless, compared to other potentials and methods, the prediction accuracies of our potentials are somewhat higher. The asymmetric *Ez* potential of Schramm & DeGrado on lipid-accessible residues yields an accuracy of 72.8%, while the sequence-based state-of-the-art methods OCTOPUS^40^ and the meta-server TopCons^41^ (both of which contain a machine learning component) predicts OPM topologies for 72.1% and 74.8% of the protein chains. For β-barrel proteins, our potentials predict OPM topologies for 91.7% of the proteins for lipid-accessible and lipid-inaccessible residues and 90.6% for lipid-accessible residues only. We believe that this value is higher for β-barrels because the asymmetry in the potentials is more pronounced than for α-helical proteins. For comparison, the asymmetric potential of Lin & Liang achieves 82.3% and sequence-based BOCTOPUS^42^ predictions achieve 71.5% as compared to OPM.

However, a truly fair comparison between structure-based and sequence-based methods is difficult, because the latter run on individual chains while the former benefit from the interconnection of the chains that constitute the protein structure. Further, our potential is trained on data similar to what OPM contains; while the protein embedding might be different, the topology is taken from OPM. Moreover, there is likely overlap between the training databases for the sequence-based methods and what OPM contains.

### Pore-facing residues in β-barrels can have sizable contribution to folded state in the membrane

Similar to Lin’s findings^28^, our β-barrel potential for lipid-accessible residues shows an energetic barrier for outer membrane proteins to spontaneously insert into an asymmetric membrane, especially for polar and charged residues and His and Trp^28^. Interestingly, our profiles show that lipid-inaccessible and therefore pore-facing residues have much more favorable free energy profiles, even for most hydrophobic residues (Fig. 2). We computed the scores for lipid-accessible and lipid-inaccessible residues separately for each protein in our databases (Fig. 5B and Supplementary Figure S3) and summed them up to obtain a total score. We find that lipid-inaccessible residues contribute on average 19.6% to the overall free energy score of α-helical proteins, and 40.7% for β-barrels, which for the latter is more than twice as high as previously suggested^28^. Further, Lin *et al*. found that for OmpLA lipid-facing residues contribute 5x more to the overall insertion process than pore-facing residues, meaning insertion is largely driven by the lipid-facing residues^28^. Our value for OmpLA (right panel in Fig. 5B) is in excellent agreement with Lin’s findings: their contribution of pore-facing residues is 16.67% while ours is 16.95%. These data show that MP insertion and stabilization have a sizable contribution from pore-facing residues, while lipid-accessible residues drive the insertion.

**Figure 6:**
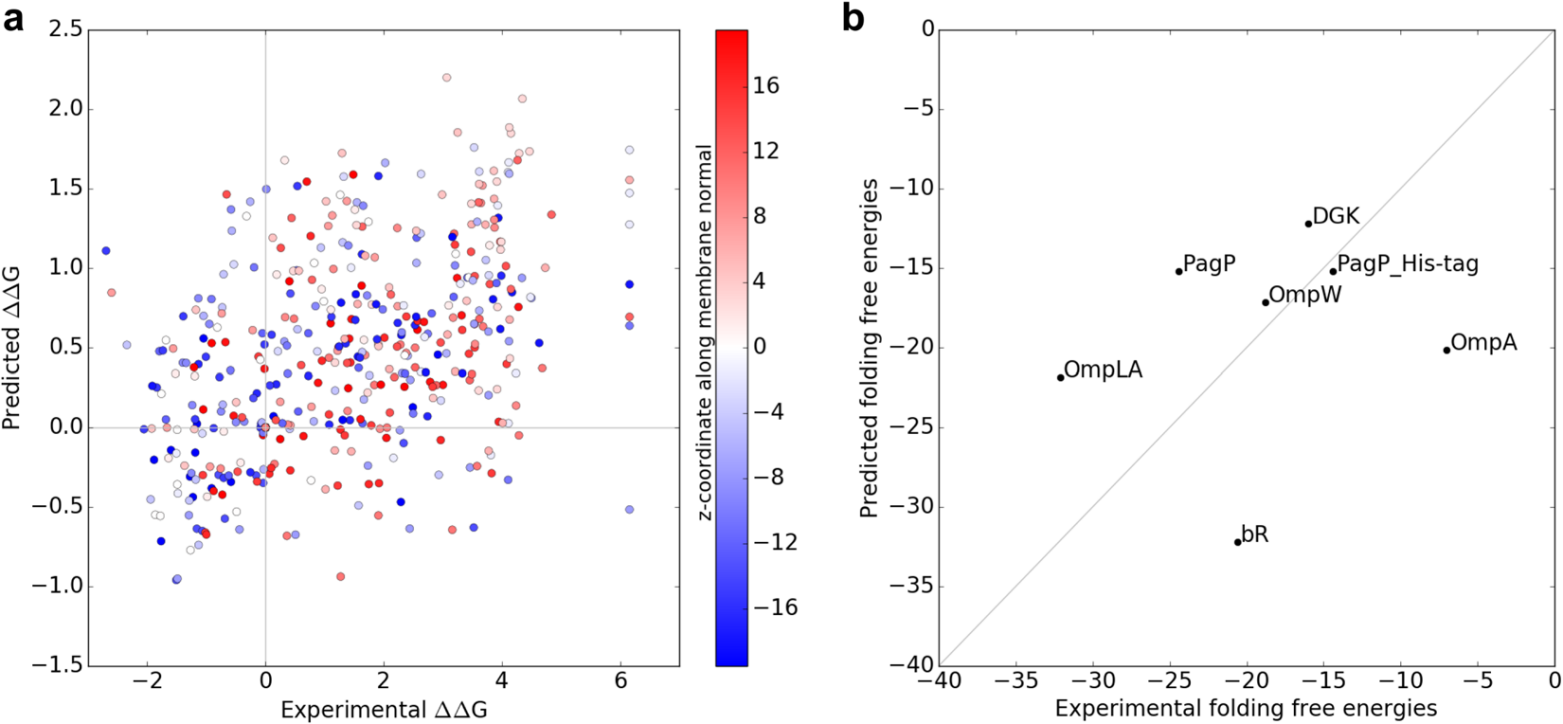
(**A**) Correlation between predicted and experimental ∆∆Gs for CLS (the C-terminal portion of L-Selectin, values were taken from Fig. 2 in Fleishman’s paper^12^) depending on the depth in the membrane with red being the external side and blue being the cytoplasmic/periplasmic side of the membrane. The Pearson correlation coefficient is 0.455 for this α-helical membrane protein, which is at the upper limit of what other methods predict on a membrane protein database^43^. (**B**) Correlation between predicted and experimentally determined folding free energies for five β-barrels and two α-helical proteins. Values were taken from Fleming’s review^44^ and only the middle value for OmpA was taken (see Table 1 in reference^44^). The Pearson correlation coefficient is 0.197.

Further, we compared experimentally determined ∆∆Gs of mutation as well as experimentally derived folding free energies with predicted values from our membrane potentials. Figure 6A shows the correlation between experimental and predicted ∆∆Gs of mutation for the CLS peptide (the C-terminal portion of L-Selectin, as shown in Fig. 2A of reference^12^), using our α-helical potential for lipid-accessible residues. While the Pearson correlation coefficient for these 455 data points appears with 0.455 relatively low, it is at the upper limit of what other ∆∆G prediction methods currently attain for MPs^43^. This is encouraging because most of the other methods are machine learning techniques specifically trained for ∆∆G prediction^43^. The low correlation may be attributed to differences between the dsTβL and our potential (Fig. 3), possibly from different interactions that are taken into account. While our profile is derived from folded proteins with both short- and long-range interactions, dsTβL is experimentally derived from a single helix with short-range interactions to neighboring residues along the helix. Further, mutation into a dissimilar amino acid might change the insertion depth in the experiment which is not taken into account or can even be easily measured.

Few experimentally derived folding free energy values exist in the literature^44^ to which we can compare our predicted scores. Figure 6B shows a correlation for five β-barrel MPs and two α-helical proteins. While the Pearson correlation coefficient is with 0.197 low and the actual values differ in most cases, the trends are similar. The low correlation likely stems from the fact that experimentally measured values (i.e. protein stability) are influenced by a number of noncovalent atomistic interactions that are not taken into account in insertion profiles.

### Implicit membrane potentials can be used to optimize protein embedding in the membrane

**Figure 7:**
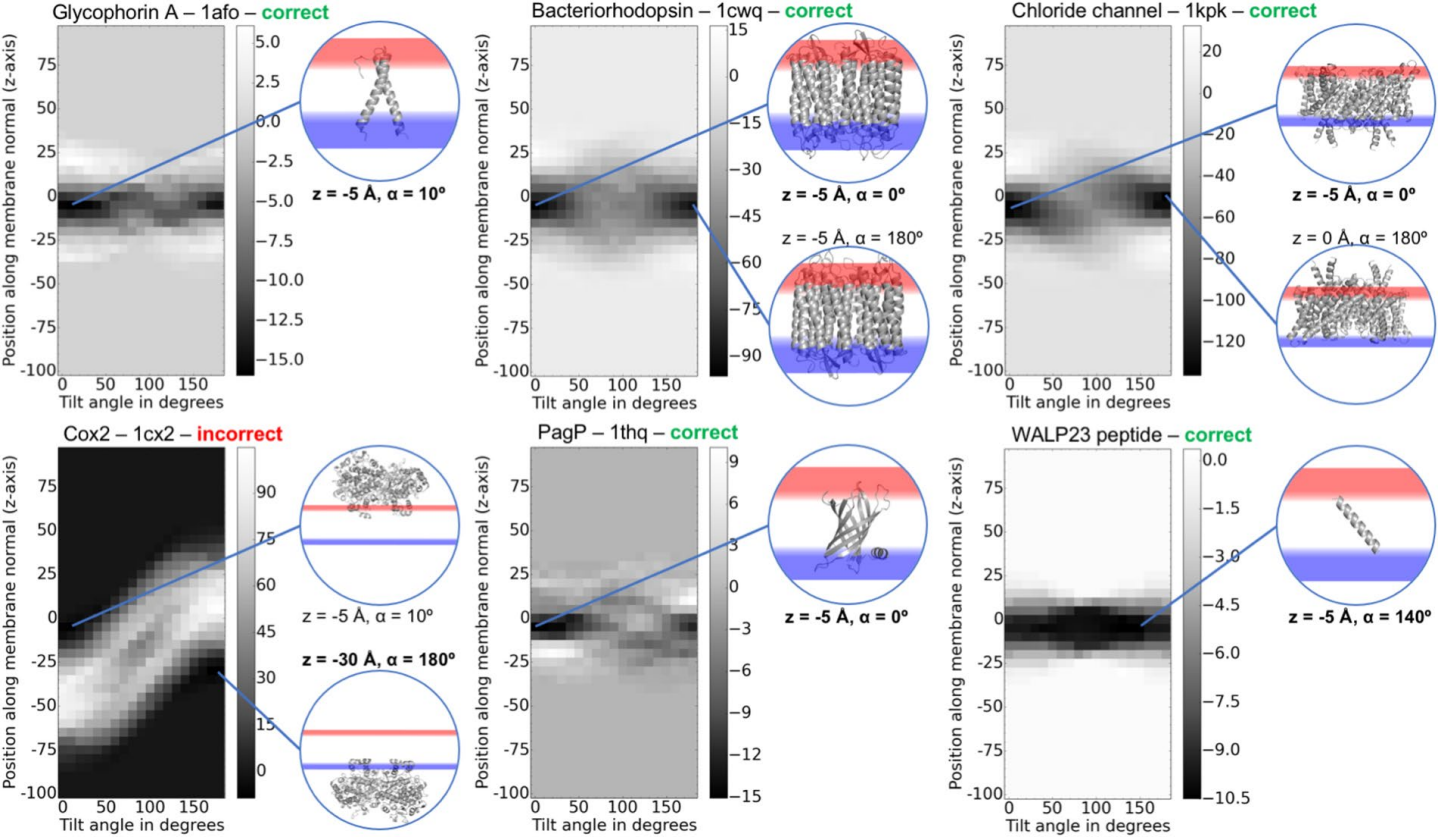
Energy profiles for different depths and tilt angles inside and outside the membrane. The native position at (0, 0) is the protein embedding from PDBTM with the topology from the OPM database. Note that at this position, the protein can already be tilted with respect to the membrane normal. The protein tilt angles at the native position are the following: Glycophorin A = 11.7°, Bacteriorhodopsin = 0°, Chloride channel = 10.7°, Cox2 = NA, PagP = 29.0°, WALP23 = 1.6°. Low energy conformations are shown in the pictures with membrane planes in red being the outer leaflet and blue being the inner leaflet. z-axis and tilt angles are shown for the low energy conformations; the lowest energy embedding parameters for the predicted topologies are highlighted in bold. Correct predictions are highlighted in green, the incorrect one (Cox2) in red.

Our implicit membrane potentials can be used to score membrane depths (z-axis) and tilt angles – we show heatmaps from 2D energy profiles for six proteins in Fig. 7. From the native position with z = 0 and tilt angle = 0 (embedding from PDBTM but topology from OPM, see database creation in Methods), each protein was translated out of the membrane by 100 Å, then scored for each position with a translation along the membrane normal in 5 Å steps and tilt angles around the x-axis in 10° steps. As shown in Fig. 7, five out of six proteins (except Cox2) have the lowest energies for orientations close to the native position. Since the potentials for lipid accessible residues are almost symmetric for α-helical proteins, flipped orientations also have low scores in many cases. For glycophorin A, bacteriorhodopsin and the chloride channel homologue, we see an energy barrier for tilt angles around 90°, which is expected. For the membrane-associated protein Cox2 the orientation on the inner leaflet of the membrane is slightly more favorable than the one on the outer leaflet by about 1.5 score points. PagP has an amphipathic helix that anchors it to the membrane and which results in an asymmetric energy profile. Hence, the local minimum at a tilt angle of 180° isn’t as deep as in other examples. The WALP23 peptide (which was modeled from sequence with tools described in^45^) has a wide energy minimum at various tilt angles in the membrane with a global minimum at 140°. This is in excellent agreement with findings from DeGrado and co-workers^25^.

## Conclusion

Here we presented implicit membrane potentials derived from databases of α-helical and β-barrel MPs. The potentials were derived under the assumption of membrane asymmetry in an identical fashion, which allows for direct comparison of the different membrane models. We found that asymmetry is much more pronounced in the β-barrel potential, in support of the vastly different membrane architecture of bacterial gram negative outer membranes, whose outer leaflet is mainly composed of lipopolysaccharide, with a highly charged inner core headgroup region. Our potentials recapitulate protein topology from the OPM database for ~80% of the α-helical proteins and > 90% of β-barrels. We further use these potentials to optimize protein embedding in the membrane as a function of membrane depth and tilt angle, where we recapitulate correct protein embedding for 5 out of 6 examples. The implicit potentials were also used to estimate the contribution of lipid-inaccessible residues to MP insertion and stability; it was found that the contribution to the overall score in α-helical proteins is ~20%, whereas pore-facing residues contribute over 40% in β-barrels, which is considerably higher than previously suggested^28^. Correlating predicted ∆∆Gs of mutation with experimental values from the literature on the α-helical C-terminal tail of L-Selectin (CLS), we achieve a correlation coefficient of 0.455, which is at the upper limit of what is currently achieved with other, specifically trained methods. Our implicit potentials can be used to improve energy functions in computational modeling tools such as the Rosetta software suite^8,9,45,46^ or MD simulation approaches^47–50^, especially accounting for asymmetry that is inherent in membrane bilayers. Possible applications of these potentials are manifold, and we have shown a few use cases here. They range from optimizing membrane embedding, identifying membrane topology, prediction of ∆∆Gs of mutation and scoring mutations for protein design to computing MP insertion energies, structure prediction, and protein-protein docking.

## Methods

### Database generation

All MP chains were downloaded from the PDBTM database^5^; α-helical bundles and membrane β-barrels were treated separately. While it would be preferable to derive membrane potentials for a variety of different membrane types, the number of proteins in each membrane type are still too small to derive reliable potentials (Supplementary Table 1). We therefore opted to differentiate by protein type, as almost all β-barrel structures are located in the bacterial outer membrane that contains an outer LPS leaflet, whereas the vast majority α-helical proteins are located in other types of bilayers. The chains were filtered with the PISCES culling server^51^ at a sequence similarity cutoff of 30% for α-helical proteins and 50% for membrane β-barrels, to account for fewer available structures of the latter. The Protein Data Bank files were culled at a resolution better than 3 Å, at an R-factor cutoff of 0.3, sequence lengths between 40 and 10,000 residues, non-Xray entries were included, Cα-only entries were excluded, and PDBs were culled by chain. We then removed electron microscopy structures with the labels “EM” or “ELEC”. We initially downloaded the structure files only from the OPM database^30,52^, because OPM uses protein topology to orient the proteins in the membrane with in/out orientation. However, we realized that protein embedding in the bilayer seems to be generally better for structures from the PDBTM database (i.e. fewer loop residues in the membrane, symmetric embedding for symmetric structures), even though the in/out orientation for those might be different from the one in OPM. We therefore flipped the PDBTM structures with inconsistent in/out topology by 180° around the x-axis (which is perpendicular to the membrane normal) to match the topology in OPM, while retaining protein embedding (depth and tilt angle) from PDBTM. We then cleaned the structures from ligands, co-factors and other hetero atoms and generated Rosetta span files from the embedded structures as described previously^45^. We further used the Rosetta *mp_lipid_acc* application on the input structures to classify residues as lipid-accessible or -inaccessible^35^.

### Histograms of amino acid occurrences, free energy profiles and fitting functions

Histograms were created by counting the occurrences of each amino acid with the computed lipid-accessibility, based on the coordinate of the Cα atom, along the membrane normal (z-axis) in bins of 3 Å width (Supplementary Figures S1 and S2). Total residue profiles were created by summing the occurrences for lipid-accessible and lipid-inaccessible residues. Fits for the total residue profiles were created with single or double-Gaussian mixture models:

1 $$\text{single}\,\text{Gaussian}\,\text{model}:{F}_{G}(z)=a+b\cdot \exp [{\frac{-(z-c)}{2{d}^{2}}}^{2}]$$

2 $$\begin{array}{c}\text{double}{\textstyle \text{-}}\text{Gaussian mixture}\,\text{model}:{F}_{dG}(z)=a+b\cdot \exp [\frac{-{(z-c)}^{2}}{2{d}^{2}}]+e\cdot \exp [\frac{-{(z-f)}^{2}}{2{g}^{2}}]\end{array}$$

Starting parameters for the fits can be estimated based on the histograms, where *a* is the constant background, *b* and *e* are the peak heights above the background, *c* and *f* are the central values, and *d* and *g* are the standard deviations. Final fit parameters are given in Supplementary Tables 2 and 3.

A window of three bins was run over the amino acid histograms and resulting values were converted into free energies (per amino acid per bin) using the inverse Boltzmann relationship with ${N}_{{aa},{bin}}$ being the residue counts of each amino acid per bin.

3 $${\rm{\Delta }}{G}_{aa,bin}=kT\,\mathrm{ln}\,\frac{{N}_{aa,bin}}{{\sum }_{aa}{N}_{aa,bin}}$$

Free energy profiles were then fit with either a double-Gaussian mixture model (*F*_*dG*_), or, if a satisfactory fit could not be achieved, a 4^th^ order polynomial (*F*_*p4*_), with fitting parameters given in Supplementary Tables 4–7:

4 $${\rm{Poly}}\,4D:{F}_{p4}(z)=a\cdot {z}^{4}+b\cdot {z}^{3}+c\cdot {z}^{2}+d\cdot z+e$$

### Scoring topologies and depths and tilt angles

‘Native’ (as described in the database generation section) and inverted topologies where scored with our implicit membrane potentials. For each residue in the membrane a score was computed, depending on the lipid accessibility and the z coordinate of the Cα atom as a measure of depth. Scores were summed individually for all lipid-accessible and lipid-inaccessible residues and native and inverted topologies. The topology with the lowest score was used as final prediction.

For each protein in our datasets with native topology, the scores for lipid-accessible and lipid-inaccessible residues were investigated separately (Fig. 5B and Supplementary Figure S3). We further computed overall scores for various depths and tilt angles (Fig. 7).

### Transmembrane span lengths and tilt angles

To compute helix/strand lengths and tilt angles (Supplementary Figure S4), we first used DSSP^53^ for identification of secondary structure. From the secondary structure elements, transmembrane spans were identified as such, if the first and last residues were on opposite sides of the membrane center plane (negative or positive z-axis). This definition ensures that secondary structure elements longer than required to span the membrane are included in the histograms. Tilt angles were computed from the center-of-masses of the (i − 1)^st^, i^th^, and (i + 1)^st^ residues of the starting and end residue of the TM spans with respect to the membrane normal. Bin widths were one residue for the transmembrane span length and 5° for the tilt angles.

### Data availability statement

The mathematical forms of the potentials are described in the Supplement to this article. PDB codes for the databases are given in the Supplement.

## Electronic supplementary material

Supplementary Information
