# Supplementary material for: Statistically derived asymmetric membrane potentials from α-helical and β-barrel membrane proteins
Source: Sci Rep. 2018 Mar 13;8:4446. doi: 10.1038/s41598-018-22476-6 (PMC5849751; doi:10.1038/s41598-018-22476-6)
Supplement: Supplementary file 1 — Supplementary Information [file 41598_2018_22476_MOESM1_ESM.pdf]

Supplementary Material for:

Statistically derived asymmetric membrane potentials from α-helical and β-barrel membrane proteins

Julia Koehler Leman[1,2], Richard Bonneau[1,2,3], Martin Ulmschneider[4]

[1] Center for Computational Biology, Flatiron Institute, Simons Foundation, New York, 10010 NY
[2] Department of Biology, Center for Genomics and Systems Biology, New York University, New York, 10003 NY
[3] Department of Computer Science, New York University, New York, 10012 NY
[4] Medical School, University of Exeter, Exeter EX1 2LU, UK

Supplementary Methods:

PDB codes of the 239 α-helical membrane proteins in our database:
1AFO, 1C17, 1FDM, 1H2S, 1JB0, 1KF6, 1KQF, 1M0K, 1M56, 1NKZ, 1OKC, 1ORS, 1P49, 1PPJ, 1RZH, 1U19, 1U7G, 1XIO, 1XRD, 1ZZA, 2A65, 2BHW, 2BL2, 2BS2, 2CFQ, 2FYU, 2H88, 2IH3, 2J58, 2J7A, 2JLN, 2K73, 2K9J, 2K9P, 2K9Y, 2KLU, 2KNC, 2KOG, 2KS9, 2KSD, 2KSE, 2KSF, 2L0J, 2L2T, 2L35, 2L9U, 2LCK, 2LOM, 2LOR, 2LOS, 2LOT, 2LP1, 2LZL, 2M67, 2M7G, 2MFR, 2MIC, 2MKV, 2MMU, 2MN6, 2MOF, 2MPN, 2MXB, 2N2A, 2N4X, 2NA8, 2NDJ, 2NQ2, 2NRG, 2QTS, 2R9R, 2VPZ, 2WDQ, 2WSW, 2XFN, 2XOV, 2YEV, 2Z73, 2ZXE, 3AR4, 3AYF, 3B9W, 3C02, 3CX5, 3D31, 3DDL, 3DH4, 3EGW, 3G6B, 3GIA, 3H90, 3K06, 3K3F, 3KLY, 3LDC, 3M73, 3MP7, 3NE2, 3NE5, 3NYM, 3PCV, 3QAP, 3QE7, 3RKO, 3RLF, 3RQW, 3S8G, 3TDS, 3TUI, 3TX3, 3UG9, 3VMA, 3VW7, 3W4T, 3WAJ, 3WFD, 3WMG, 3WO6, 3WU2, 3WXW, 3ZE3, 3ZOJ, 4A01, 4AL0, 4BEM, 4BVN, 4C7R, 4COF, 4DVE, 4DX5, 4ENE, 4EZC, 4G7V, 4GC0, 4GX0, 4HFI, 4HUQ, 4HYJ, 4I0U, 4IFF, 4IKV, 4J05, 4K1C, 4KPP, 4KYT, 4LP8, 4LXJ, 4M5B, 4MBS, 4MES, 4MND, 4MRS, 4N6H, 4N7W, 4NV5, 4O6M, 4O6Y, 4O93, 4OGQ, 4P02, 4P79, 4PD6, 4PGR, 4PHZ, 4PL0, 4Q4H, 4QND, 4QTN, 4QUV, 4R0C, 4RDQ, 4RI2, 4RNG, 4RP9, 4RYO, 4TQ3, 4U4T, 4U9N, 4UC1, 4UMW, 4UVM, 4V1G, 4WD8, 4WFE, 4X5M, 4X89, 4XES, 4XK8, 4XNV, 4XP9, 4XTL, 4XU4, 4YMK, 4YMU, 4YSX, 4Z3N, 4ZP0, 4ZR1, 4ZW9, 5A1S, 5AJI, 5AWW, 5AX0, 5AYN, 5AZB, 5B0W, 5B1A, 5BW8, 5BZ3, 5C6O, 5C6P, 5C78, 5CKR, 5DQQ, 5DWY, 5EDL, 5EIK, 5EKE, 5EZM, 5FXB, 5G28, 5G2C, 5G2D, 5HK1, 5HYA, 5I20, 5I32, 5ID3, 5IU4, 5IWS, 5J4I, 5KBW, 5KUK, 5SVK, 5SYT, 5T1A, 5TCX, 5TIS, 5U09

PDB codes of the 96 β-barrel membrane proteins in our database:
1A0T, 1EK9, 1FEP, 1KMO, 1P4T, 1QD6, 1QJ8, 1QJP, 1UUN, 1UYN, 1WP1, 1XKW, 1YC9, 2ERV, 2F1V, 2FGQ, 2GR8, 2GUF, 2HDI, 2LHF, 2LME, 2MAF, 2MPR, 2POR, 2VDF, 2W16, 2WJR, 2X27, 2X55, 2X9K, 2Y2X, 2YNK, 3AEH, 3BS0, 3CSL, 3DWO, 3DZM, 3EFM, 3FHH, 3FID, 3GP6, 3KVN, 3O44, 3PGU, 3PRN, 3QQ2, 3QRA, 3RFZ, 3SY7, 3SY9, 3SYB, 3SZV, 3T0S, 3V8X, 3VY8, 3W9T, 3X2R, 4AFK, 4B7O, 4C00, 4C69, 4CU4, 4D5B, 4E1S, 4E1T, 4FQE, 4FRX, 4FSO, 4FSP, 4FUV, 4GEY, 4K3C, 4K7R, 4MEE, 4MT4, 4N75, 4Q35, 4QL0, 4RDR, 4RJW, 4RL8, 4RLC, 4Y25, 5DL5, 5DL6, 5DL7, 5DL8, 5FOK, 5FP1, 5FR8, 5FVN, 5IUY, 5IVA, 5IXM, 5LDV, 7AHL

Supplementary Table 1:
Number of redundant membrane protein structures for different types of membrane bilayers. Counts were taken from the OPM website (http://opm.phar.umich.edu/localization.php).

membrane type[a]	***pro/eu***[b]	***#prot***[c]	***#sol P***[d]	***#MPs***[e]	*α*[f]	*β*[g]
archaebacterial membrane	pro	94	7	87	87	-
bacterial gram-neg IM	pro	494	94	400	400	-
bacterial gram-neg OM	pro	227	31	196	7	**189**
bacterial gram-pos OM	pro	10	9	1	-	**1**
bacterial gram-pos plasma membrane	pro	144	47	97	97	-
sum[h]					591	190
cytoplasmic granule membrane	-	1	1	-	-	-
secreted	-	981	938	43	35	8[i]
thylakoid membrane	-	42	14	28	28	-
undefined	-	118	113	5	3	2
viral membrane	-	91	64	27	27	-
sum					93	10
chloroplast IM	eu	2	2	-	-	-
chloroplast OM	eu	2	2	-	-	-
ER membrane	eu	209	118	91	91	-
endosome membrane	eu	56	47	9	9	-
eukaryotic plasma membrane	eu	845	427	418	418	-
golgi membrane	eu	35	35	-	-	-
lysosome membrane	eu	23	20	3	3	-
mitochondrial IM	eu	80	38	42	42	-
mitochondrial OM	eu	33	15	18	10	8
nuclear IM	eu	5	5	-	-	-
nuclear OM	eu	6	6	-	-	-
peroxisome membrane	eu	11	11	-	-	-
vacuole membrane	eu	28	18	10	10	-
vesicle membrane	eu	22	21	1	1	-
sum					584	8

[a] – different membrane types; OM = outer membrane; IM = inner membrane
[b] – eukaryotic or prokaryotic membrane type
[c] – total number of proteins in that type of membrane according to the OPM database
[d] – number of membrane-associated, but not transmembrane, proteins in that membrane type, i.e. these proteins are soluble
[e] – number of membrane proteins in that membrane
[f] – how many of the membrane proteins are α-helical
[g] – how many of the membrane proteins are β-barrels
[h] – sum of α-helical and β-barrels for this class of membranes
[i] – secreted β-barrel MPs are lysins for which the barrel consists of multiple chains

Supplementary Figure S1:
Amino acid occurrences for α-helical proteins, considering lipid-accessible and lipid-inaccessible residues separately. Counts were averaged over 3Å bins along the membrane normal.

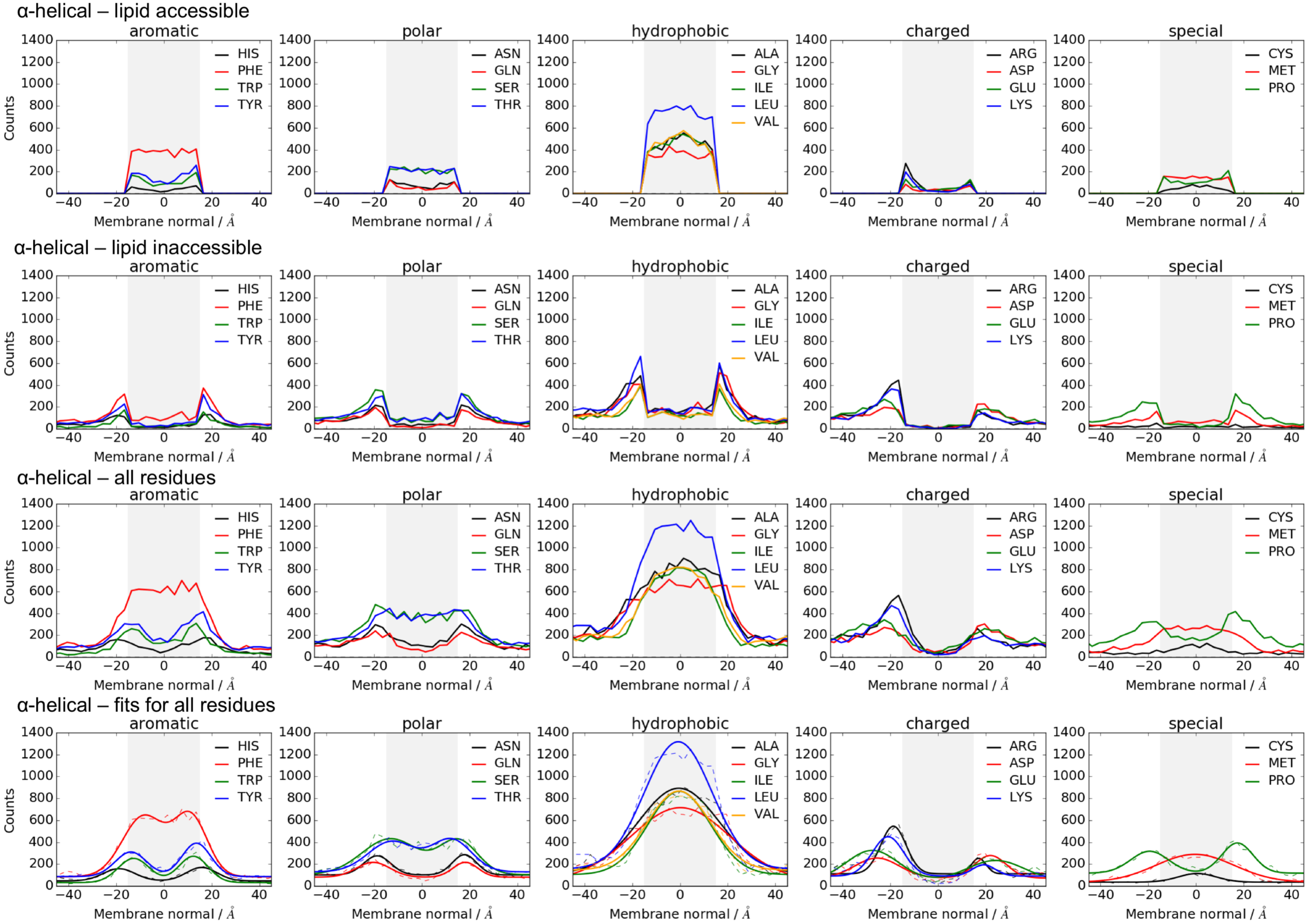

Supplementary Table 2:
Fitting parameters for total counts for α-helical proteins (see Supplementary Figure S1).

amino acid	function	a	b	c	d	e	f	g
**HIS**	dgaussian	45.9426	111.8477	-18.7311	6.6892	123.2780	15.8104	6.4044
**PHE**	dgaussian	84.0570	553.0258	-8.4815	8.8068	544.4241	11.1981	6.9751
**TRP**	dgaussian	30.6708	221.0713	-12.6161	6.5237	240.7302	12.3390	6.1409
**TYR**	dgaussian	85.9869	224.5648	-13.8022	6.5776	304.8907	13.5983	6.0264
**ASN**	dgaussian	100.7527	174.4990	-18.7619	5.6477	184.9904	17.7994	5.0672
**GLN**	dgaussian	80.0819	135.5682	-20.4137	7.0684	135.7772	18.1724	5.2534
**SER**	dgaussian	103.6965	327.8742	-13.1740	11.9214	307.0977	15.3144	7.9998
**THR**	dgaussian	128.8560	278.1981	-12.6311	9.8470	293.4502	12.3766	8.3310
**ALA**	gaussian	113.2601	779.1964	-0.4875	16.9416			
**GLY**	gaussian	47.6585	667.4080	0.1731	20.7867			
**ILE**	gaussian	103.4647	761.5255	-0.2855	13.0411			
**LEU**	gaussian	153.2482	1163.4179	-0.7183	14.1093			
**VAL**	gaussian	143.6374	723.0583	-0.4278	13.1572			
**ARG**	dgaussian	110.7870	434.7847	-18.6558	5.3030	141.9693	17.0798	3.0190
**ASP**	dgaussian	72.7777	182.3676	-25.4930	9.2611	205.5760	21.9673	6.5730
**GLU**	dgaussian	46.0040	279.2446	-27.4897	10.9056	187.4936	24.1953	11.8811
**LYS**	dgaussian	90.6900	359.0646	-20.8885	6.5083	103.0028	19.1817	4.7431
**CYS**	gaussian	34.9335	78.7127	0.4979	7.9379			
**MET**	gaussian	34.2900	254.8837	-0.4510	16.3663			
**PRO**	dgaussian	117.5398	199.7092	-19.4333	7.9062	275.4525	17.3003	6.5076

Supplementary Figure S2:

Amino acid occurrences for β-barrel proteins, considering lipid-accessible and lipid-inaccessible residues separately. Counts were averaged over 3Å bins along the membrane normal.

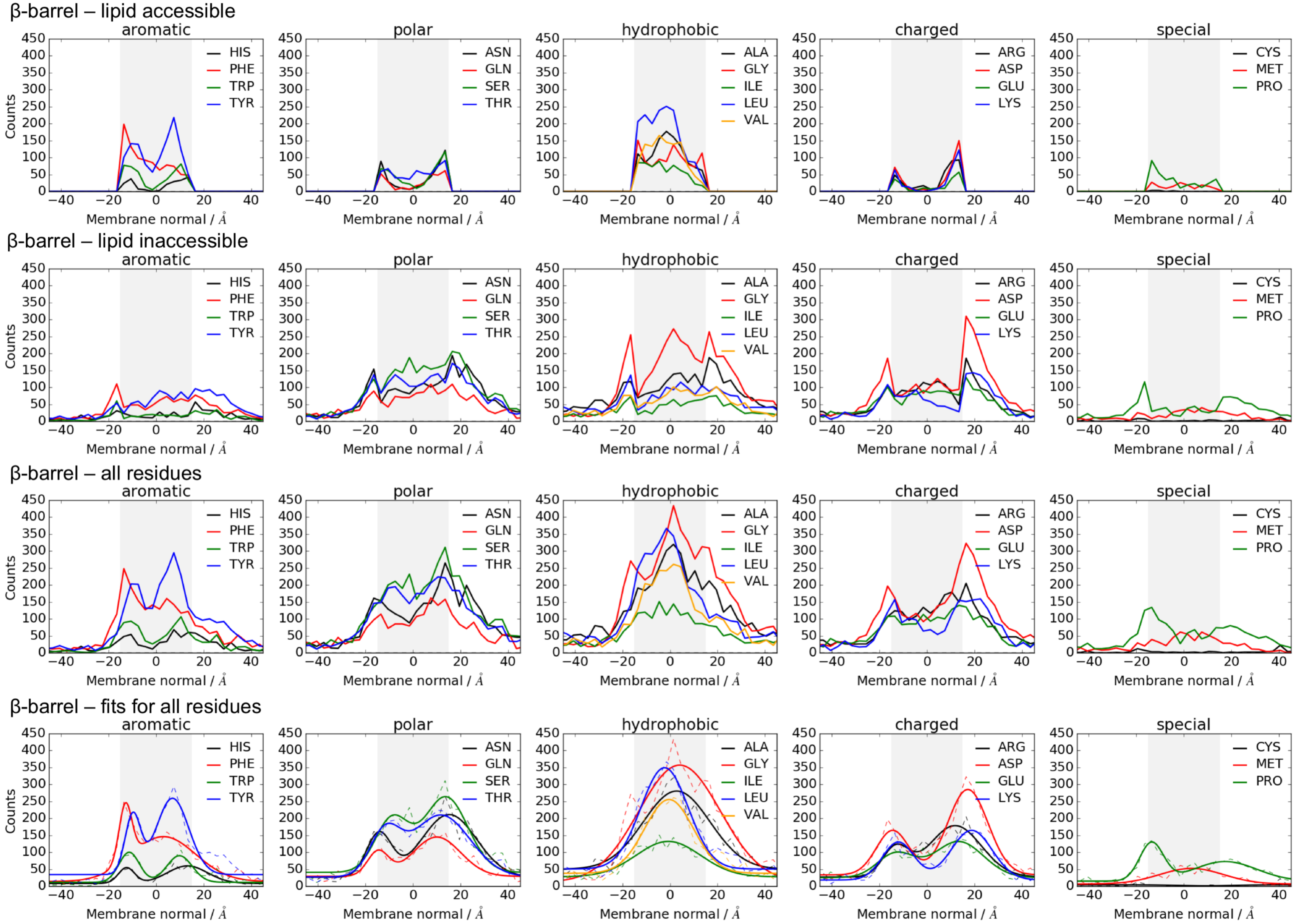

Supplementary Table 3:
Fitting parameters for total counts for β-barrel proteins (see Supplementary Figure S2).

amino acid	function	a	b	c	d	e	f	g
**HIS**	dgaussian	8.2031	46.0225	-12.3860	3.4095	51.5503	13.1994	8.4881
**PHE**	dgaussian	13.5052	165.4294	-12.8650	2.6833	132.6475	3.1051	13.6012
**TRP**	dgaussian	11.3502	88.6820	-11.2581	4.2830	78.5192	9.8533	5.3468
**TYR**	dgaussian	34.1224	172.0264	-9.7808	-3.2421	225.0861	6.9283	6.8709
**ASN**	dgaussian	26.2122	127.4087	-14.3430	5.2280	184.9654	15.1838	11.5617
**GLN**	dgaussian	28.9380	71.9467	-14.7575	4.4234	115.9907	9.9989	10.0199
**SER**	dgaussian	40.6483	156.7841	-8.8986	7.0755	222.5365	13.7595	9.2514
**THR**	dgaussian	27.1084	124.4057	-11.8590	6.3334	182.7258	11.6498	12.3484
**ALA**	gaussian	49.8598	230.6812	2.8176	14.0284			
**GLY**	gaussian	11.7367	345.2370	4.0755	17.1572			
**ILE**	gaussian	27.5348	104.5430	-0.6901	13.5805			
**LEU**	gaussian	50.5373	298.7281	-2.2565	9.9534			
**VAL**	gaussian	38.1035	217.5332	-0.3895	10.2598			
**ARG**	dgaussian	27.0737	84.7174	-13.1119	5.4989	151.5175	11.9019	10.9967
**ASP**	dgaussian	33.2685	131.1659	-14.3802	6.8511	251.8404	17.2180	8.4928
**GLU**	dgaussian	25.0175	71.0348	-13.0992	7.7363	106.7172	13.3075	10.4151
**LYS**	dgaussian	18.1530	111.8809	-11.9914	6.6853	146.4727	18.8114	8.6094
**CYS**	gaussian	4.0620	-3.0216	8.8661	11.8573			
**MET**	gaussian	6.1678	46.1242	3.2229	13.4698			
**PRO**	dgaussian	14.8918	111.5543	-13.5661	5.0001	57.7180	17.3520	13.2913

Supplementary Table 4:
Fitting parameters for implicit membrane potential for α-helical proteins and lipid accessible residues (see Figure 2).

amino acid	function	a	b	c	d	e	f	g	residual
HIS	dgaussian	0.644	0.379	-0.864	4.032	0.309	-3.647	10.069	0.041
PHE	dgaussian	1.900	-114.339	2.124	-556.724	112.093	-0.120	803.289	0.132
TRP	dgaussian	0.669	-0.488	-14.624	6.484	-0.570	16.959	8.314	0.135
TYR	dgaussian	-432.871	433.008	-23.494	1319.332	0.370	0.289	4.741	0.141
ASN	dgaussian	-226.712	0.274	1.306	3.618	227.260	1.558	453.920	0.213
GLN	dgaussian	-0.802	-0.246	14.552	4.608	1.783	3.644	21.540	0.327
SER	dgaussian	0.019	-0.052	-7.004	3.275	-1143217.656	1225.720	-92.327	0.110
THR	dgaussian	0.005	-0.041	-14.337	7.356	-1143217.656	1225.720	-92.327	0.082
ALA	dgaussian	-0.225	-0.288	1.987	10.600	-0.037	-0.603	0.786	0.091
GLY	dgaussian	4.162	-115.350	0.028	-429.832	110.873	-7.915	883.013	0.281
ILE	dgaussian	-1.199	0.945	-22.213	19.828	1.069	27.319	14.420	0.059
LEU	dgaussian	-0.764	0.242	-22.321	7.317	0.109	15.827	5.989	0.055
VAL	dgaussian	304.943	-0.056	1.562	-5.057	-305.432	-0.408	414.496	0.111
ARG	dgaussian	-0.151	1.371	0.492	6.720	0.528	19.864	11.672	0.323
ASP	dgaussian	0.631	0.502	-6.791	3.694	0.472	4.589	5.246	0.237
GLU	dgaussian	0.127	0.171	-2.667	3.262	0.853	0.108	9.902	0.317
LYS	dgaussian	0.004	1.489	1.154	7.412	0.404	16.750	3.356	0.279
CYS	dgaussian	17.414	-0.760	1.001	-10.268	-16.018	-0.723	-188.362	0.207
MET	dgaussian	2.191	-113.071	-2.883	-594.978	111.104	2.494	778.256	0.119
PRO	dgaussian	-0.169	0.074	0.236	-4.892	0.583	-4.213	-14.948	0.164

Supplementary Table 5:
Fitting parameters for implicit membrane potential for α-helical proteins and lipid inaccessible residues (see Figure 2).

amino acid	function	a	b	c	d	e	f	g	residual
**HIS**	dgaussian	0.287	0.560	-6.816	6.725	0.233	5.254	8.905	0.215
**PHE**	dgaussian	0.012	-0.035	21.620	0.582	-0.294	3.191	10.943	0.207
**TRP**	poly4d	0.000	0.000	-0.001	-0.016	0.558	-	-	0.539
**TYR**	dgaussian	-0.178	0.556	-2.669	12.983	0.191	-4.527	2.596	0.382
**ASN**	dgaussian	0.096	0.302	7.629	3.717	0.285	-7.442	-6.288	0.135
**GLN**	dgaussian	0.059	0.816	-0.722	10.498	-0.232	5.819	-2.551	0.150
**SER**	dgaussian	-0.154	0.053	-1.588	4.882	-1143217.656	1225.720	-92.327	0.254
**THR**	dgaussian	-0.179	0.129	-14.087	5.644	-1143217.656	1225.720	-92.327	0.432
**ALA**	dgaussian	1070.802	-1071.379	1.351	-832.579	-1143217.656	1225.720	-92.327	0.229
**GLY**	dgaussian	2584.551	-2585.073	0.967	-1315.588	-1143217.656	1225.720	-92.327	0.407
**ILE**	dgaussian	-0.101	-0.035	21.620	0.582	-0.343	-2.398	8.978	0.395
**LEU**	dgaussian	-0.178	-0.376	-2.325	18.002	-1143217.656	1225.720	-92.327	0.087
**VAL**	dgaussian	-0.195	-0.035	21.620	0.582	-0.211	-3.281	7.380	0.440
**ARG**	dgaussian	-0.418	1.098	10.002	7.174	1.410	-4.749	5.593	0.724
**ASP**	dgaussian	-0.016	0.520	-9.198	5.595	1.103	4.249	5.842	0.637
**GLU**	dgaussian	0.228	0.687	-2.003	6.339	0.226	11.252	1.890	0.338
**LYS**	dgaussian	-1.412	0.525	2.994	1.709	2.642	2.018	15.301	0.707
**CYS**	dgaussian	1826.803	-0.035	21.620	0.582	-1826.134	-0.184	690.803	0.353
**MET**	dgaussian	676.000	-0.035	21.620	0.582	-675.991	0.443	504.612	0.255
**PRO**	dgaussian	-0.096	0.567	5.524	3.684	0.406	-5.893	-8.485	0.269

Supplementary Table 6:
Fitting parameters for implicit membrane potential for β-barrel proteins and lipid accessible residues (see Figure 2).

amino acid	function	a	b	c	d	e	f	g	residual
**HIS**	dgaussian	-543.314	1.281	-2.018	3.081	544.076	-49.107	1520.739	0.513
**PHE**	dgaussian	-0.571	-0.136	6.414	-3.884	0.756	18.227	15.817	0.113
**TRP**	dgaussian	2268.535	1.011	-1.128	4.095	-2268.798	-1.895	-826.541	0.483
**TYR**	dgaussian	0.331	-0.730	-9.552	6.838	-0.951	7.359	5.304	0.296
**ASN**	dgaussian	-141.565	141.560	-6.652	320.865	1.051	-2.304	5.443	0.288
**GLN**	dgaussian	-0.155	0.408	-18.012	27.657	0.881	-3.437	4.754	0.386
**SER**	dgaussian	-0.487	0.599	-17.677	20.128	0.775	1.105	-4.328	0.106
**THR**	dgaussian	-4.835	115.484	-10.740	368.628	-110.555	15.296	-872.002	0.304
**ALA**	dgaussian	-1.328	1.342	15.826	10.693	1.167	-14.487	7.966	0.147
**GLY**	dgaussian	-126.817	-0.331	1.144	-4.359	126.700	0.856	281.733	0.140
**ILE**	poly4d	0.000	0.000	0.004	0.021	-0.195	-	-	0.362
**LEU**	dgaussian	-2.126	2.523	22.202	16.175	1.437	-17.468	9.936	0.317
**VAL**	dgaussian	-0.762	1.295	17.600	7.458	1.328	-29.431	12.095	0.267
**ARG**	dgaussian	-0.251	0.899	-7.883	7.971	0.648	0.861	3.973	0.507
**ASP**	dgaussian	-0.524	0.492	-15.813	3.457	1.951	-1.495	6.420	0.236
**GLU**	dgaussian	-12999.587	13006.329	-1525.155	47781.041	1.171	-1.646	7.672	0.817
**LYS**	dgaussian	-0.616	1.104	-5.885	12.758	1.499	-0.598	4.393	0.676
**CYS**	-	-	-	-	-	-	-	-	-
**MET**	poly4d	0.000	0.000	0.002	-0.007	0.649	-	-	0.538
**PRO**	dgaussian	-0.143	0.617	7.577	12.261	0.226	1.922	5.407	0.139

Supplementary Table 7:
Fitting parameters for implicit membrane potential for β-barrel proteins and lipid inaccessible residues (see Figure 2).

amino acid	function	a	b	c	d	e	f	g	residual
**HIS**	dgaussian	0.623	0.284	8.639	-2.752	0.423	-3.317	4.590	0.400
**PHE**	dgaussian	0.308	-0.470	-7.232	12.398	0.454	-4.604	5.944	0.110
**TRP**	poly4d	0.000	0.000	0.000	0.005	0.884	-	-	0.512
**TYR**	dgaussian	0.681	-0.649	6.671	18.437	-0.119	-10.570	2.213	0.080
**ASN**	dgaussian	-5.284	116.313	0.107	347.070	-111.165	36.742	-2262.392	0.322
**GLN**	dgaussian	37.569	-0.158	9.557	-4.053	-37.546	-9.669	-405.899	0.184
**SER**	dgaussian	-0.015	-0.379	12.327	9.330	-0.410	-7.446	6.651	0.163
**THR**	dgaussian	-0.108	-0.180	10.886	4.677	-0.281	-8.918	4.673	0.100
**ALA**	dgaussian	2.222	110.312	-0.144	814.458	-112.775	8.550	-538.112	0.246
**GLY**	dgaussian	-0.029	-0.621	-3.255	20.295	0.212	-10.070	3.782	0.083
**ILE**	dgaussian	0.197	0.218	-10.009	1.849	0.110	-6.568	7.466	0.260
**LEU**	dgaussian	2.279	110.370	-25.815	2914.210	-112.718	-0.068	-471.648	0.530
**VAL**	dgaussian	2.298	110.388	-5.320	-17095.160	-112.698	2.350	-359.740	0.426
**ARG**	dgaussian	-0.066	-0.134	-4.760	-7.661	-1143217.656	1225.720	-92.327	0.334
**ASP**	dgaussian	-598.378	0.171	9.882	1.778	598.245	-1.780	560.561	0.140
**GLU**	dgaussian	2.784	-113.807	-1.779	-560.687	110.981	3.888	806.902	0.176
**LYS**	dgaussian	-0.111	0.507	10.255	3.227	0.358	2.911	-3.717	0.192
**CYS**	-	-	-	-	-	-	-	-	-
**MET**	dgaussian	0.889	-0.327	-1.472	3.873	-0.379	9.121	4.385	0.324
**PRO**	dgaussian	0.036	0.629	-2.988	5.123	0.513	10.726	5.112	0.238

Supplementary Figure S3:

Free energy scores for the native position (position (0, 0) in Figure 6) of each protein in the database: The scores for the lipid-accessible residues is shown in orange, for lipid-inaccessible residues in blue and the total scores are shown as '–'. (A) Scores for all 239 proteins in our α-helical membrane protein database. Lipid-inaccessible residues contribute on average about 19.57% to the total score – these residues are most often buried in the protein interior. (B) Scores for all 96 proteins in our β-barrel database. Lipid-inaccessible residues contribute on average 40.70% to the overall score. Since lipid-inaccessible residues mostly face the aqueous pore in β-barrels, the contribution of pore-facing residues to overall insertion and stability is considerably higher than was previously suggested by Liang et al.[1]. However, there is excellent agreement between the values suggested for OmpLA (gray box): Liang et al. estimated that lipid-facing contribute 16.67% to overall insertion, while our predicted value for OmpLA is 16.95%.

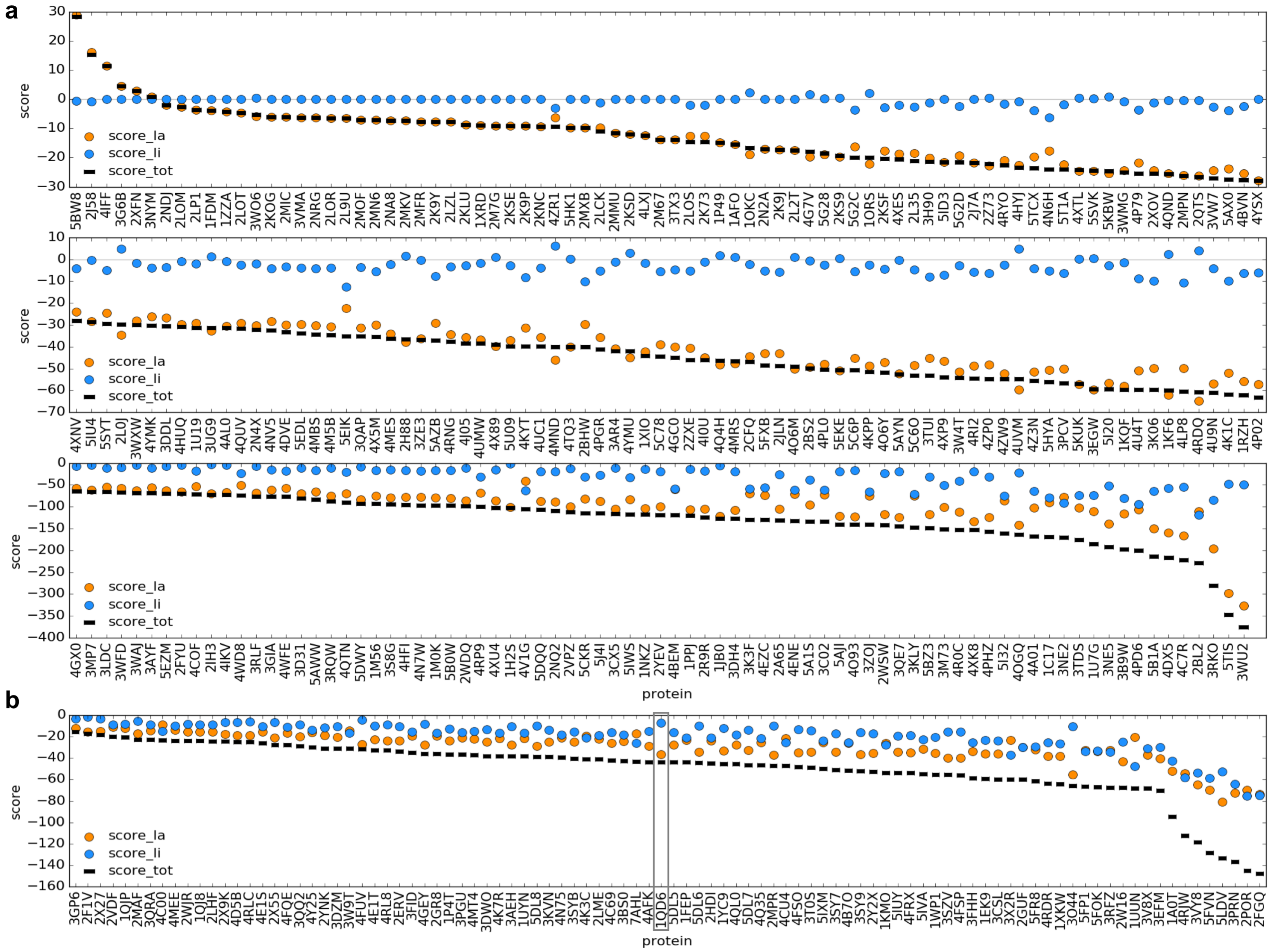
a
b
score
protein
score_la
score_li
score_tot
5BW8 2J58 4IFF 3G6B 2XFN 3NYM 2NDJ 2LOM 2LP1 1FDM 1ZZA 2LOT 3WO6 2KOG 2MIC 3VMA 2NRG 2LOR 2L9U 2MOF 2MN6 2NA8 2MKV 2MFR 2K9Y 2LZL 2KLU 1XRD 2M7G 2KSE 2K9P 2KNC 4ZR1 5HK1 2MXB 2LCK 2MMU 2KSD 4LXJ 2M67 3TX3 2LOS 2K73 1P49 1AFO 1OKC 2N2A 2K9J 2L2T 4G7V 5G28 2KS9 5G2C 1ORS 2KSF 4XES 2L35 3H90 5ID3 5G2D 2J7A 2Z73 4RYO 4HYJ 5TCX 4N6H 5T1A 4XTL 5SVK 5KBW 3WMG 4P79 2XOV 4QND 2MPN 2QTS 3VW7 5AX0 4BVN 4YSX
4XNV 5IU4 5SYT 2L0J 3WXW 4YMK 3DDL 4HUQ 1U19 3UG9 4AL0 4QUV 2N4X 4NV5 4DVE 5EDL 4MBS 4M5B 5EIK 3QAP 4X5M 4MES 2H88 3ZE3 5AZB 4RNG 4J05 4UMW 4X89 5U09 4KYT 4UC1 4MND 4TQ3 2BHW 4PGR 3AR4 4YMU 1XIO 5C78 4GC0 2ZXE 4I0U 4Q4H 4MRS 2CFQ 5FXB 2ILN 4O6M 2BS2 4PL0 5EKE 5C6P 4KPP 4O6Y 5AYN 5C6O 3TUI 4XP9 3W4T 4RI2 4ZP0 4ZW9 4UVM 4Z3N 5HYA 3PCV 5KUK 3EGW 5I20 1KQF 4U4T 3K06 1KF6 4LP8 4RDQ 4U9N 4K1C 1RZH 4P02
4GX0 3MP7 3LDC 3WFD 3WAJ 3AYF 5EZM 2FYU 4COF 2IH3 4IKV 4WD8 3RLF 3GIA 4WFE 3D31 5AWW 3RQW 4QTN 5DWY 1M56 3S8G 4HFI 4N7W 1M0K 5B0W 2WDQ 4RP9 4XU4 1H2S 4V1G 5DQQ 2NQ2 2VPZ 5CKR 5J4I 3CX5 5IWS 1NKZ 2YEV 4BEM 1PPJ 2R9R 1JB0 3DH4 3K3F 4EZC 2A65 4ENE 5A1S 3C02 5AJI 4O93 3ZOJ 2WSW 3QE7 3KLY 5BZ3 3M73 4R0C 4XK8 4PHZ 5I32 4OGQ 4A01 1C17 3NE2 3TDS 1U7G 3NE5 3B9W 4PD6 5B1A 4DX5 4C7R 2BL2 3RKO 5TIS 3WU2
3GP6 2F1V 2X27 2VDF 1QJP 2MAF 3QRA 4C00 4MEE 2WJR 1QJ8 2LHF 2X9K 4D5B 4RLC 4E1S 2X55 4FQE 3QQ2 4Y25 2YNK 3DZM 3W9T 4FUV 4E1T 4RL8 2ERV 3FID 4GEY 2GR8 1P4T 3PGU 4MT4 3DWO 4K7R 3AEH 1UYN 5DL8 3KVN 4N75 3SYB 4K3C 2LME 4C69 3BS0 7AHL 4AFK 1QD6 5DL5 1FEP 5DL6 2HDI 1YC9 4QLO 5DL7 4Q35 2MPR 4CU4 4FSO 3T0S 5IXM 3SY7 4B7O 3SY9 2Y2X 1KMO 5IUY 4FRX 5IVA 1WP1 3SZV 4FSP 3FHH 1EK9 3CSL 3X2R 2GUF 5FR8 4RDR 1XKW 3O44 5FP1 5FOK 3RFZ 2W16 1UUN 3V8X 3EFM 1A0T 4RIW 3VY8 5FVN 5LDV 3PRN 2POR 2FGQ

Supplementary Figure S4:
Histograms for TM span lengths (left) and tilt angles (right) for α-helical (top) and β-barrel (bottom) membrane proteins derived from our databases show that statistics agree well with previous analyses. When deriving statistics on the α-helical and β-barrel databases, we also investigated TM span lengths and tilt angles for individual secondary structure elements (as opposed to tilt angles of entire proteins). For the α-helical bundles in our database, TM spans were on average 26.0 ± 7.9 residues in length with an average tilt angle of 22.9 ± 12.7°. These values are in excellent agreement with previous analyses[2,3]. For the β-barrels in our database, TM spans were on average 12.9 ± 4.1 residues in length with an average tilt angle of 37.3 ± 9.6°. From these values, an average membrane thickness can be estimated, which would be 35.9 Å for the thickness derived from α-helical MPs (assuming a 1.5 Å rise per residue) and 36.0 Å for the thickness derived from β-barrel MPs (assuming a 3.5 Å rise per residue). These values, however, are derived under the assumption that the entire TM span is located in the membrane, which doesn't always hold true, especially not for many outer membrane β-barrels that have long strands sticking into the oligosaccharide region of the LPS outer membrane leaflet[4].

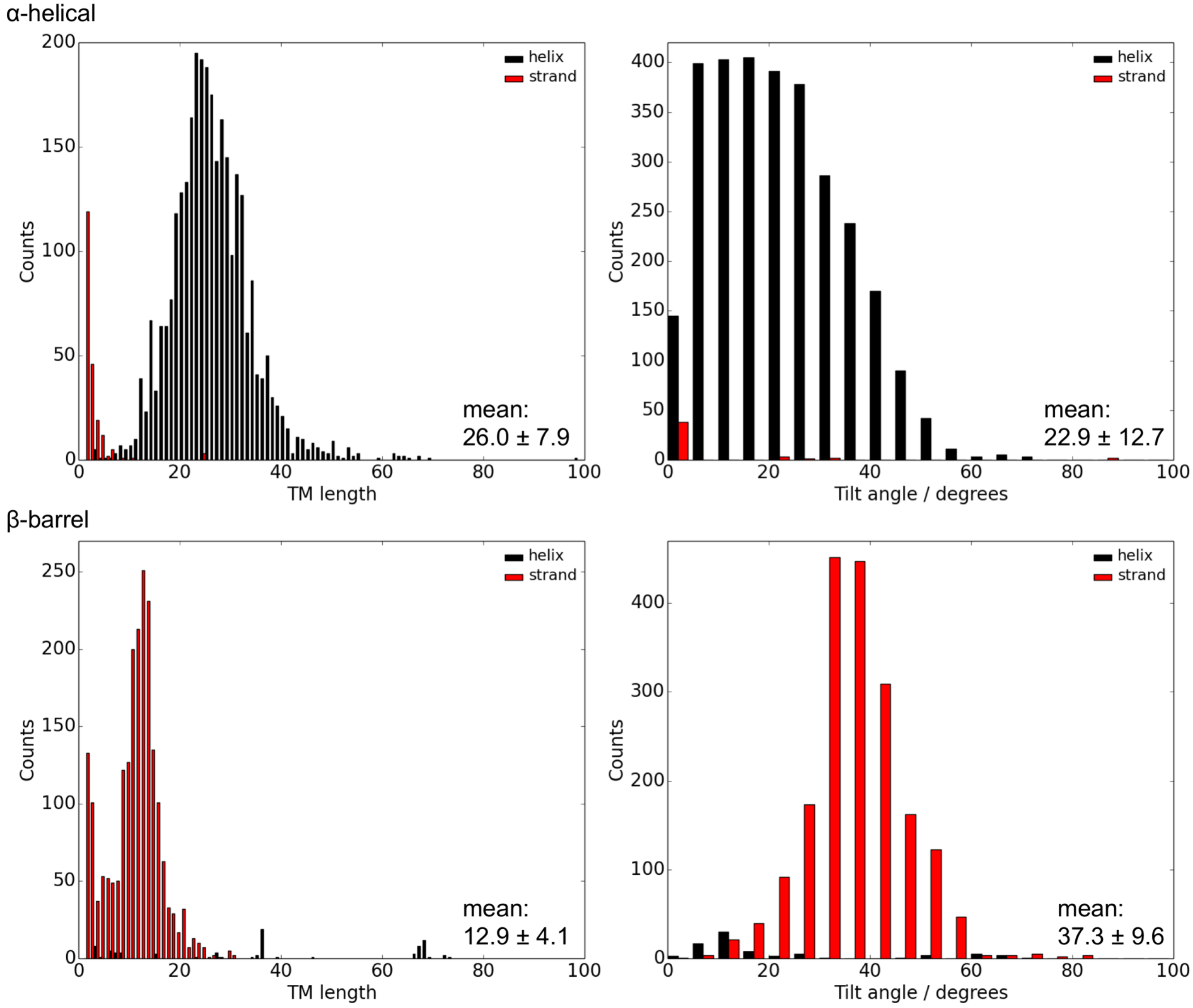

References:

1. Lin, M., Gessmann, D., Naveed, H. & Liang, J. Outer membrane protein folding and topology from a computational transfer free energy scale. *J. Am. Chem. Soc.* **138,** 2592–2601 (2016).
2. Ulmschneider, M. B., Sansom, M. S. P. & Di Nola, A. Properties of integral membrane protein structures: Derivation of an implicit membrane potential. *Proteins Struct. Funct. Genet.* **59,** 252–265 (2005).
3. Bowie, J. U. Helix packing in membrane proteins. *J. Mol. Biol.* **272,** 780–789 (1997).
4. Pavlova, A., Hwang, H., Lundquist, K., Balusek, C. & Gumbart, J. C. Living on the edge: Simulations of bacterial outer-membrane proteins. (2016). doi:10.1016/j.bbamem.2016.01.020
